# Targeted delivery of paclitaxel drug using polymer-coated magnetic nanoparticles for fibrosarcoma therapy: in vitro and in vivo studies

**DOI:** 10.1038/s41598-023-30221-x

**Published:** 2023-02-23

**Authors:** Rusul Al-Obaidy, Adawiya J. Haider, Sharafaldin Al-Musawi, Norhana Arsad

**Affiliations:** 1 Applied Sciences Department/Laser Science and Technology Branch, University of Technology, Baghdad, Iraq; 2 College of Food Sciences, Al-Qasim Green University, Babylon, Iraq; 3 Photonics Technology Laboratory, Department of Electrical, Electronic and Systems Engineering, Faculty of Engineering and Built Environment, Universiti Kebangsaan Malaysia UKM, 43600 Bangi, Malaysia

**Keywords:** Biotechnology, Nanobiotechnology

## Abstract

Fibrosarcoma is a rare type of cancer that affects cells known as fibroblasts that are malignant, locally recurring, and spreading tumor in fibrous tissue. In this work, an iron plate immersed in an aqueous solution of double added deionized water, supplemented with potassium permanganate solution (KMnO_4_) was carried out by the pulsed laser ablation in liquid method (PLAIL). Superparamagnetic iron oxide nanoparticles (SPIONs) were synthesized using different laser wavelengths (1064, 532, and 266 nm) at a fluence of 28 J/cm^2^ with 100 shots of the iron plate to control the concentration, shape and size of the prepared high-stability SPIONs. The drug nanocarrier was synthesized by coating SPION with paclitaxel (PTX)-loaded chitosan (Cs) and polyethylene glycol (PEG). This nanosystem was functionalized by receptors that target folate (FA). The physiochemical characteristics of SPION@Cs-PTX-PEG-FA nanoparticles were evaluated and confirmed by Fourier transform infrared spectroscopy (FTIR), scanning electron microscopy (SEM), transmission electron microscopy (TEM), X-Ray diffraction (XRD), atomic force microscopy (AFM), and dynamic light scattering (DLS) methods. Cell internalization, cytotoxicity assay (MTT), apoptosis induction, and gene expression of SPION@Cs-PTX-PEG-FA were estimated in fibrosarcoma cell lines, respectively. In vivo studies used BALB/c tumor-bearing mice. The results showed that SPION@Cs-PTX-PEG-FA exhibited suitable physical stability, spherical shape, desirable size, and charge. SPION@Cs-PTX-PEG-FA inhibited proliferation and induced apoptosis of cancer cells (P < 0.01). The results of the in vivo study showed that SPION@Cs-PTX-PEG-FA significantly decreased tumor size compared to free PTX and control samples (P < 0.05), leading to longer survival, significantly increased splenocyte proliferation and IFN-γ level, and significantly decreased the level of IL-4. All of these findings indicated the potential of SPION@Cs-PTX-PEG-FA as an antitumor therapeutic agent.

## Introduction

Numerous medical researchers have evaluated cancer treatment over the past 20 years, and polymeric nanoparticles have proven to be quite effective at delivering drugs to specific locations^1^. Although a wide range of nanomaterials are used in biomedical applications. The magnetic nanoparticles (MNPs) appear to have the highest degree of success potential. Many different types of studies have been done on the usage of SPIONs to treat cancer cells in vitro^2^. Superparamagnetic iron oxide (Fe_3_O_4_) nanoparticles have emerged as sensitive therapeutic carriers for various applications among the wide variety of MNPs^3^. As a consequence, the purpose of this study is to give a condensed description of the most significant components of SPIONs in targeted drug delivery systems for cancer treatment. Laser ablation in liquid (LAIL) is a physical and chemical process that involves the ablation of a solid target in liquid media by using a laser beam with a high degree of purity. It is a relatively simple and flexible technique for NP formation without the use of any surfactant and counter-ion^4^. Ablative nanoparticles (NPs), in contrast to those produced by chemical synthesis, comprise nothing but the target material and the liquid (i.e., they do not include any contaminants) and can be used in different applications such as sensors, storage energy, solar cell, photocatalysis, biosensors^5–7^. Accordingly, the LAIL technique is a simpler way to collect NP because the particles are preserved in their colloid state rather than being absorbed by the chamber walls or the substrate^8^. In addition, selecting the appropriate kind of polymer and concentration of stabilizing agent based on their ability to wet the surfaces of the particles and create a barrier may help prevent agglomeration of nanoparticles, which is another method for preventing the accumulation of nanoparticles. Therefore, Dadashi S, et al. in 2015 synthesized iron oxide NPs by (LAIL) in DDW and acetone using water with many conventional organic liquids^9^, but Valery A. Svetlichny in 2019 obtained spherical NPs of about 2–80 nm containing Fe_3_O_4_ α-Fe_2_O_3_, γ-Fe_2_O_3_, and FeO with pulsed focused radiation from an Nd: YAG laser at 1064 nm in DDW^10^. In 2020 Maurizio Muniz-Miranda et al. studied the core–shell Fe_3_O_4_@Au NPS by the basal wavelength (1064 nm)^11^, and Adawiya et al. in the 2022 formulation of curcumin in the folate-functionalized polymeric coated Fe_3_O_4_@Au core–shell nanosystem for targeting breast cancer therapy (MDA-MB-231) and normal (MCF 10A) cell lines, achieved using the Pulsed Laser Ablation in Liquid (PLAL) procedure by the 530 nm wavelength with various laser fluence (1.8, 2.3, and 2.6) J/cm^2^^12^. In this work, the potassium permanganate (KMnO_4_) used is a versatile oxidizing agent for studying the oxidation kinetics of many organic substrates. KMnO_4_ is intrinsically unstable and slowly but observably decomposing. In neutral or slightly alkaline solutions in the dark, decomposition is immeasurably slow. However, Fe_3_O_4_–KMnO_4_ NPs have different applications, such as water treatment, due to their large surface area^13,15^.

**Scheme 1:**
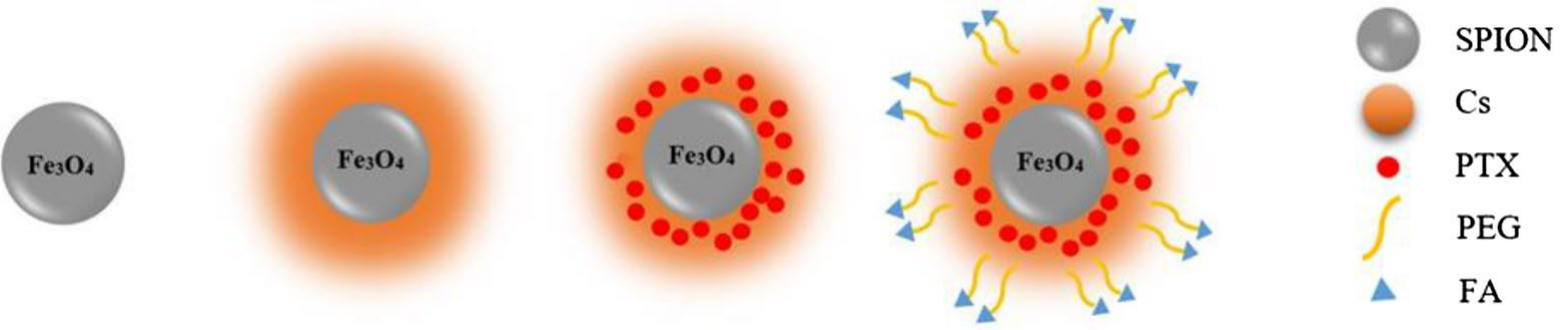
Schematic of the preparation and synthesis of SPION@Cs-PTX-PEG-FA NPs. Abbreviations: SPION, PEG, Cs, PTX, and FA.

Cancer is one of the leading causes of mortality worldwide, particularly with fibrosarcoma that can develop at any age, but most often in those aged 20–60 years^16^. They are predominantly located in deep soft tissue or adjacent to bones. Traditional treatments such as surgery, radiation, and chemotherapy serve as the primary treatments. Among them, chemotherapy is still the most commonly used^17–22^. Fibrosarcoma is recognized as both an important and well-known kind of cancer. Almost a hundred new cases of this malignancy are documented every year. Fibrosarcoma is distinguished from other types of cancer by its resistance to radiation therapy and chemotherapy, as well as by the high frequency with which it returns after treatment^23^. As a result, it is essential to investigate novel approaches that are capable of enhancing the therapy of this particular tumor form. The removal of the tumor by surgical procedures is the primary therapeutic option for fibrosarcoma. Surgery is often followed by a course of radiation treatment to reduce the probability of cancer returning after removal, and this is the case regardless of the tumor grade^24^. However, the significant constraints of chemotherapy drugs such as weak bioavailability, negligible circulating half-life, low stability, and toxicity to normal cells or healthy tissue decrease the efficacy of this approach. Cancer-targeted drug delivery using a nanotechnology approach does not cause noticeable side effects unlike traditional chemotherapeutic routes. However, the development of efficient targeted drug delivery systems has remained a major concern^25,26^. The Pacific Yew L plant mainly produces the chemical known as paclitaxel (PTX) when removed. When administered systemically, mainly by injection into a vein, PTX has shown notable activity in clinical trials against various types of tumors, including lung, cervical, breast, and pancreatic cancer, head and neck carcinoma, advanced ovarian carcinoma, cancer, and acute leukemia^27–29^. This activity has been observed when the drug was administered. Various solid tumors and hematologic malignancies are extensively treated with this molecule, but its clinical use is limited by different factors such as low water solubility and poor bioavailability^30^. In addition, most chemotherapeutic agents induce apoptosis in cancer cells and severely damage normal host cells. Combined drug delivery systems, such as polymeric-metallic nanocarriers, can develop antitumor efficacy through targeted tumor delivery, bioavailability, and decreased toxicity of free PTX^31^. Meanwhile, the intrinsic fluorescence property of this molecule serves as a fluorescence probe to facilitate the efficient investigation of drug-loaded NP uptake. This molecule has also been reported to exert various effects on signaling molecules, such as activation of apoptotic pathways, induction of cell cycle cessation, and down-regulation of angiogenesis-associated gene expression^32–34^. The antioxidant property of PTX is well defined by its ability to intercept mutagenic/carcinogenic reactive oxygen species (ROS; for example, peroxides, hydroxyl radicals, superoxide anions, and nitrite radicals)^35^. In particular, the reduced systemic bioavailability of PTX void due to its poor absorption, inappropriate dispersity in vivo, rapid metabolism, and rapid systemic elimination lead to its limited anticancer activity and unsatisfactory results in cancer therapy^36^. Furthermore, the efficiency of a chemotherapeutic agent such as PTX can be improved by formulating novel systems such as NPs for its delivery^37^. Meanwhile, the application of a nanosized core–shell-based drug delivery system using natural polymers and biocompatible metallic NPs appears to be an encouraging and reliable approach to cancer treatment, with improved targeting anticancer efficiency and reduced toxic side effects^38–41^. PTX encapsulation in NPs based on polymer metals is attracting more attention because it enhances internalization/localization and consequently induces cytotoxicity and apoptosis in cancer cells^,42–45^. Among metallic NPs, magnetic iron oxide has a very noticeable capability to improve efficient drug delivery systems including easy synthesis with certain controllable characteristics, such as size, shape, magnetism, and flexibility, in the formulation of multifunctional superparamagnetic iron oxide nanoparticles (SPIONs) by different materials (e.g., biocompatible polymers, targeting ligands, drugs, fluorescence, etc.)^46^. Furthermore, drug-loaded magnetic NPs can be easily guided to the tumor region of target using an external physical magnetic force due to blood circulation. Fe_3_O_4_ NPs form large aggregates if subjected to a powerful magnetic force. The modification of SPION with some polymers develops its stability and improves the biocompatibility property due to the drug-delivery process^47^. However, man-made and natural materials have been used successfully in the process of coating SPION. The biocompatibility and biodegradability of natural materials such as polysaccharides make them particularly outstanding as building materials. Meanwhile, chitosan (Cs) has attracted a remarkable amount of attention because of its good biocompatibility, biodegradability, bioactivity, low toxicity, and reactive chemical groups, including –OH and –NH2. Given that Cs has mucoadhesive and bioadhesive properties, Cs-coated SPION has a high probability of entering normal tissues. This finding is disadvantageous for a targeted drug delivery system. Literature on adopting Cs-coated SPION as a drug-delivery nanoformulation is highly limited. PEG has wide-ranging applications within the pharmaceutical industry. The attachment of PEG to nanocarrier surfaces is known to decrease the clearance and promote water solubility. Furthermore, this polymer increases the stability of NPs and prolongs their circulation half-life in vivo by decreasing enzymatic degradation due to minimizing nonspecific interactions^48^. This polymer is highly recommended for the functionalization of drug-loaded nano-carriers by targeting molecules such as the antibodies, peptides, aptamers, or ligands of some overexpressed molecules in tumor cells (e.g., transferrin and folate (FA)). The FA molecule can bind to the FA receptor (FR) and simplify the transfer of FA-decorated nano-carriers through receptor-mediated endocytosis into the cytosol^48^. In the present study, we prepared a magnetic nano-sized carrier for PTX-targeted delivery. SPIONs were synthesized by LAIL at different laser wavelengths (1064, 532, 266 nm) and fluence of 28 J/cm^2^ by 100 shots. A KMnO_4_ solution was added to the liquid. Cs-magnetic NPs (SPION@Cs) were fabricated by the reverse micro-emulsion method. Simultaneously, PTX was added to the SPION-Cs solution to form SPION@Cs -PTX. The FA-PEG combination that formed owing to electrostatic linkage was conjugated onto the SPION@Cs from the PEG direction through the amidation reaction (Scheme1). After the synthesis and characterization of SPION@Cs-PTX-PEG-FA composition, its potential for the loading and delivery of PTX to treat the BALB/c mouse fibrosarcoma cell line WEHI-164 were evaluated. The normal mouse embryonic fibroblast cell line MEF was used to compare the effect of the prepared nano-formulation for normal and cancer cells. This study was extended to in vivo conditions to treat BALB/c mouse by the WEHI-164 fibrosarcoma cell line WEHI-164. Tumor growth delay time, survival of treated mice, and their immune response were further studied.

## Materials and methods

### Reagents

The 99.99% pure Fe plate had dimensions of 1 × 1 × 0.5 cm^3^ and was supplied from Fe plate (Sigma Aldrich Company). The purity was assessed using the device (SKYRAY EDX P 370, China). The ablation process was performed at room temperature (RT) in double deionized water (DDW) of 99.99% purity. All other reagents were purchased from Sigma–Aldrich (St. Louis, MO, USA) unless otherwise specified. Low-molecular-weight Cs were purchased from Durect Corp (AL, USA).

### Synthesis of SPION NPs

**Figure 1:**
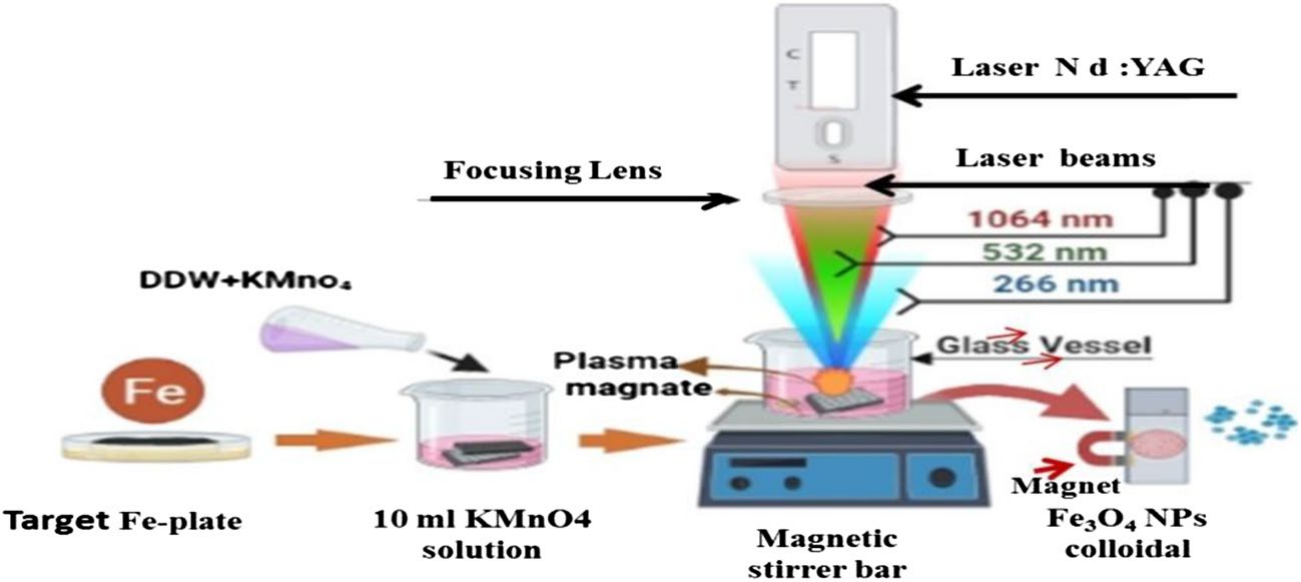
Schematic of the experimental setup for Fe_3_O_4_ NPs prepared by LAIL with different wavelengths and KMnO_4_ solution.

We dissolved 0.01 mg of KMnO_4_ in 10 ml of DDW at RT and poured it into a 100 ml beaker under stirring conditions (700 rpm). This solution was approximately 2–4 mm above the pure, polished, and cleaned Fe target as shown in Fig. 1. The Q-switching Nd: The YAG laser beam entered the vessel from above, thereby impinging perpendicularly onto the target (Fe plate) in three ways with all parts of the PLAIL system. Firstly: Focused laser beam with the fundamental wavelength at 1064 nm by positive lens (focal length 5 cm), secondly: Second Harmonic Generation (SHG) wavelength at 532 nm, and several ways: Third Harmonic Generation (THG) wavelength at 266 nm (without focusing) laser beam on the target surface. To achieve uniform ablation, also to avoid the absorption of the laser beam by the newly formed NPs and persistent microbubbles, hot plate magnetic stirring was used. The output pulsed laser for all above ways has pulsed duration of 10 ns, laser fluence of 28 J/cm^2^, 900 mJ laser energy, number of pulses equal 100, 3 Hz repetition rate, and beam diameter of 2 mm. This process took 20 min.

However, after the laser is placed on the Fe plate to initiate the separating process of the Fe atoms from it, the atoms should be strongly oxidized. So, Fe_3_O_4_ must predominantly be formed instead of Fe_2_O_3_ and FeO since the oxygen present in the water molecules (H_2_O) is not enough for Fe_3_O_4_ formation. For this purpose, KMnO_4_ was added to the liquid to provide enough oxygen for the Fe atoms, to obtain the Fe_3_O_4_ form predominantly in the liquid. These Fe_3_O_4_ nanoparticles produced will be rapidly precipitated by external magnetic field beside the beaker due to its super paramagnetic properties as shown in Fig. 1. Then the product was washed with DDW more than once to remove Mn and other salt residues that were formed during the reaction. The process of this reaction is described in Eq. (1).

1 $${\text{KMnO}}_{4} + {\text{Fe}} + {\text{H}}_{2} {\text{O}} \to {\text{KOH}} + {\text{MnO}}_{2} + {\text{Fe}}_{3} {\text{O}}_{4} .$$

Subsequently, the Fe_3_O_4_ NPs were collected by a magnet with high magnetic strength and washed twice with ethanol and then dried in a vacuum oven at 40 °C for 15 min.

### Preparation of super paramagnetic Cs with PTX (SPION@ Cs-PTX)

**Figure 2:**
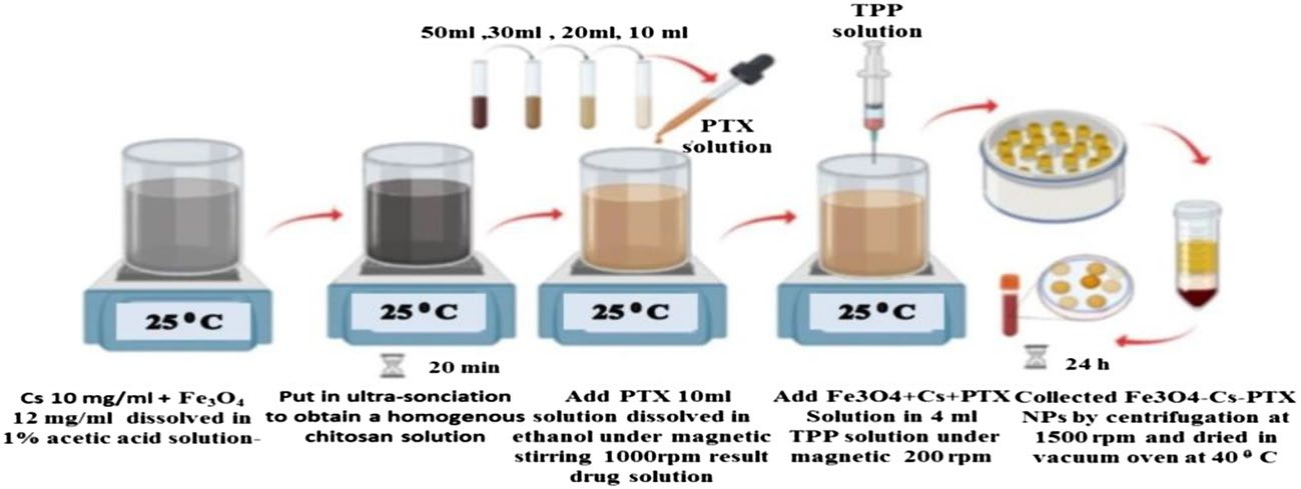
Schematic of the preparation and synthesis of SPION@Cs-PTX NPs.

Cs (10 mg/mL) and SPION (12 mg/mL) were first dissolved in 1% acetic acid aqueous solution. To prepare SPION@Cs solution consistent throughout the experiment, the resultant suspension was ultrasonically treated for 20 min. In a separate container, 20 mg of PTX was dissolved in dimethyl sulfoxide (DMSO). Then, the drug solution was added to 10 mL of the SPION@Cs solution under magnetic stirring (1000 rpm) at RT to obtain SPION@Cs-PTX solution. The SPION@Cs -PTX solution was added drop wise (using a disposable syringe with a 22-gauge needle) into 4 mL of TPP solution (2 mg/mL) under magnetic stirring (200 rpm) at RT. SPION@Cs NPs containing PTX were generated abruptly and had a Cs texture. To facilitate more cross-linking of the NPs, the PTX-Cs-SPION NP solution was agitated at 25 °C for 90 min. Afterwards, the SPION@Cs -PTX NPs were collected by centrifugation at a speed of 1500 rpm and dried in a vacuum oven at 40 °C for 24 h (Fig. 2).

### Preparation of SPION@ Cs-PTX-PEG-FA

**Figure 3:**
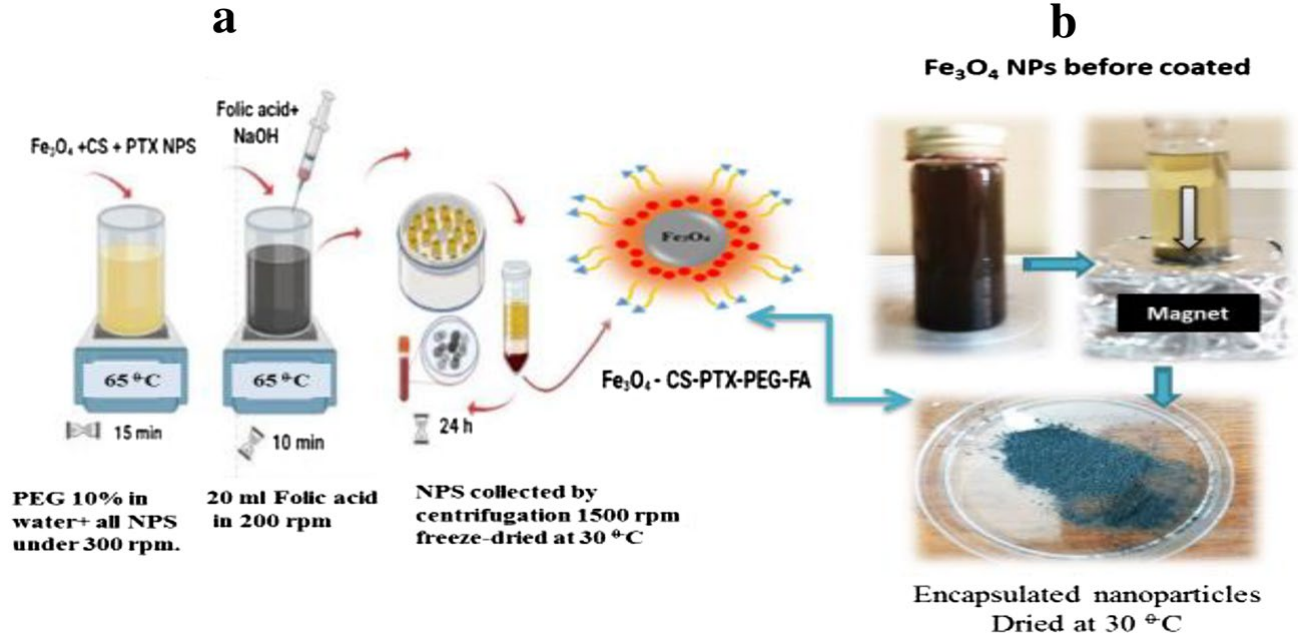
(**a**) Schematic of the preparation and synthesis of SPION@Cs-PTX-PEG-FA NPs, and (**b**) photos during their preparation in the laboratory.

First, a PEG solution containing 10% of the total water volume was prepared. The PEG solution was then stirred into the SPION@Cs-PTX mixture at a continuous magnetic stirring speed of 300 rpm for 1 h while the mixture was left at RT. The final solution of SPION@Cs-PTX-PEG-FA was spun at 200 rpm for 10 min at RT while adding 20 ml dropwise of 5 mg/ml of FA. Figure 3 shows how the different percentages of encapsulated nano- composites (SPION@Cs-PTX-PEG-FA) were collected by spinning at 1500 rpm followed by freezing at − 30 °C for 20 h.

### Characterization of magnetic composite NPs

#### UV–Vis measurement

Optical absorption was measured using a double beam UV–Vis spectrophotometry system (Aquarius 7000) within the range of 200–800 nm. Each sample was placed inside a quartz cuvette with a 10 mm optical path length as the industry standard. Spectra were taken at wavelengths between 200 and 800 nm. The absorption of the SPION before and after being coated by spectral peaks study.

#### Measurements of particle size and zeta (ζ)-potential measurements

The size, polydispersity, and zeta-potential of SPION@Cs-PTX-PEG-FA were measured with a Zetasizer Nano-ZS (Malvern Instruments, UK). After preparing a solution of nanoparticle PTX in double distilled water at 1 mg/ml, the mixture was sonicated for 15 s in an ice cold bath. At RT, every measurement of the PTX nanoformulation was performed using three independent replicates.

#### Atomic force microscopy (AFM)

The morphological characteristics of SPION@Cs-PTX-PEG-FA were determined by AFM (Dimension Icon, Bruker, USA). A drop of PTX nanoformulation solution (1 mg/ml) was located on freshly cleaved mica. After 3 min of incubation, the surface was gently washed with DDW to remove unbound NPs.

#### Transmission electron microscopy (TEM)

The interior properties of the PTX nanoformulation were determined by high-resolution TEM (JEM-2000 EXII, JEOL, Tokyo, Japan) at 120 kV. A drop of diluted PTX nanoformulation solution was placed on a 200 mesh formvar carbon-coated copper grid (TABB Laboratories Equipment).

#### Scanning electron microscopy (SEM)

We determined the element concentrations for the target Fe plate before ablation and after the laser process by energy-dispersive X-ray spectroscopy (EDX). The morphology of the PTX-loaded nanocomposite was studied by SEM (LEO 1525 field emission scanning electron microscope, Zeiss, Oberkochen, Germany). The dry PTX loaded nanocomposite was located on aluminum stubs with double-sided conducting carbon tapes and covered with a 50/50 mixture of Au/Pd. The samples were scanned at an accelerating voltage of 25 kV.

#### Fourier transform infrared (FTIR) spectroscopy

Changes in composition of various NPs were recorded on a Nicolet 6700 infrared detector (Thermo Fisher Scientific, USA). SPIONs, SPION@Cs, SPION@Cs-PEG-FA and SPION@Cs-PEG-FA were pressed with KBr to obtain the pellets at a pressure of 300 kg/cm^2^. The FTIR spectra of the above sample were obtained by averaging 32 interferograms within the range of 1000–4000 cm^–1^ with a resolution of 2 cm^–1^.

#### X-ray diffraction (XRD)

The crystal structures of SPIONs and SPION@Cs-PEG-FA were studied by XRD on a RIGAKU X-ray diffractometer (model Miniflex IL, USA) applying the CuKα as a radiation source. All samples were measured at a voltage of 30 kV, current of 25 mA, scan range of 5°–65°, and scan interval of 1°/min.

#### Vibrating sample magnetometer (VSM)

The VSM measures the magnetization of a small sample of magnetic material placed in an external magnetizing field by converting the dipole field of the sample into an AC electrical signal. The magnetometer may be used as a field measuring device by using a paramagnetic sample. A VSM system (Lakeshore 7404, USA) was utilized to evaluate the magnetic properties.

#### Drug encapsulation efficiency and drug loading content

The encapsulation efficiency was evaluated by resuspending 1 mg of PTX-loaded NP in 1 ml of phosphate buffered saline (PBS) and incubating for 2 h at 37 °C on a rocker. The amount of encapsulated PTX was assessed as the difference between the total amount of fed PTX used for nanoformulation and the free unloaded PTX. To calculate the free PTX content, the solution containing NP loaded with PTX and free PTX was dialyzed with a dialysis membrane bag against deionized water for 48 h. The amount of free PTX in the dialysis solution was evaluated by ultraviolet (UV) spectrophotometry (Infinite M 200 fluorophotometer, TECAN, USA). All experiments were performed in triplicate. Encapsulation efficiency and drug loading content were calculated using the following formula^48–51^:

2 $$Encapsulation\, efficiency \left( \% \right) = \frac{Total \,amount \,of\, drug - Free \,drug}{{Initial \,amount \,of\, drug}} \times 100,$$

3 $$Drug\,loading\,content \left( \% \right) = \frac{Total \,amount \,of\,drug - Free\,drug}{{Total\,amount\,of\,the \,nanoparticles}} \times 100.$$

#### Release profile

SPION @ Cs-PEG-FA loaded with PTX (1 mg) was resuspended in 1 ml of buffers (ranging from pH 2.0 to 8.0) and incubated for 5 h on a rocker at 37 °C. At determined time distances, the fluorescence emission intensities of the solutions were assessed at 591 nm to define the amount of released PTX. All assays were performed in triplicate. The percentage of PTX released was acquired according to the following equation:

4 $$\mathrm{R}=\frac{\mathrm{V }{\sum }_{\mathrm{i}}^{\mathrm{n}-1}{\mathrm{C}}_{\mathrm{i}}+ {\mathrm{V}}_{\mathrm{o}}{\mathrm{C}}_{\mathrm{n}}}{{\mathrm{m}}_{\mathrm{drug}}} .$$

The drug concentrations were presented using Ci and Cn, where i is the test moment as well as "n", and the drug shows the encapsulated drug mass in a nanocarrier. R is the release of the drug expressed as a percentage, V is the sampling volume, and V0 is the initial drug volume. Drug concentrations are presented as Ci and Cn, where i is the initial drug volume. The material that had become sedimentary was washed once again with double-distilled water before being suspended^52^.

### In vitro cell studies

#### Cell-culture conditions

The normal mouse embryonic fibroblast cell line MEF-1 (CRL-2214) and the BALB/c mouse fibrosarcoma cell line WEHI-164 (CRL-1751; obtained from ATCC, USA) were grown in Dulbecco's modified Eagle's medium (DMEM; GIBCO, USA). DMEM contained 10% fetal bovine serum, antibiotics (1% penicillin and streptomycin), and 200 mM glutamine (GIBCO, USA) at 37 °C in a humidified atmosphere of 5% CO_2_. Female BALB/c mice (6–8 weeks old, 20–25 g in weight) were obtained from the animal house of the Faculty of Veterinary Medicine Al-Qasim Green University. They were housed in a conventional animal facility under standard laboratory conditions^53^.

#### Cell internalization assay

Using a fluorescence microscope, the capacity of SPION@Cs-PTX-PEG-FA for cell internalization was evaluated after functionalization with fluorescein 5(6)-isothiocyanate (FITC) dye (Nikon Eclipse TE2000-U, Temecula, CA, USA). FITC-Cs was produced by connecting fluorescein isothiocyanate (5-isomer) to the Cs polymer through a reaction between the isothiocyanate group of FITC and the main amino group of CS, leading to the formation of FITC-Cs. To investigate the process of cellular internalization, FITC-SPION@Cs-PTX-PEG-FA was analyzed using WEHI-164 cell lines by observing the fluorescent emission produced by PTX inside the cell medium. Overnight, the cells were seeded onto coverslips with DMEM medium and placed on six-well plates so that they could attach. After separation, the medium was rinsed with PBS at 37 °C, and the cells were treated with FITC-SPION@Cs-PTX-PEG-FA for 4 h. Following incubation, the medium was discarded and the cells were washed with PBS three times. On a microscope slide, cover slips were placed in various positions. After 3 h of treatment with 5 g of FITC-SPION@Cs-PTX-PEG-FA, cells were examined. Subsequently, the media that contained the nanocomposite were thrown away, and the cells were rinsed with PBS.

#### Cytotoxicity assay

Cell viability was evaluated using the 3-(4,5-dimethylthiazol-2-yl)-2,5-diphenyl tetrazolium bromide (MTT) assay, which was based on the ability of mitochondrial succinate dehydrogenase in living cells to reduce yellow tetrazolium salt to violet formazan compounds. In a typical procedure, WEHI-164 and MEF-1 cells were seeded on separate 96-well sterile plates at a density of 104 cells/well in 200 µl of DMEM and allowed to adhere and grow for 24 h. The cells were then treated with a fresh medium comprising serial concentrations (0–50 μM) of PTX in the form of PTX-loaded SPION@Cs-PEG-FA and PTX void solutions for 24 and 48 h. Void NPs (SPION@Cs-PEG-FA) and medium were applied separately as positive and negative controls, respectively. Subsequently, the culture medium was removed and the cultures were incubated with 10 μl of 5 mg/ml of MTT for an additional 4 h at 37 °C followed by the addition of 200 μL of DMSO. Cell viability was determined at 545 nm using a 96-well plate reader (VictorTM X3, 2030 Multilabel Reader, PerkinElmer, Italy). The IC50 was determined by the standard curve method as the sample concentration that caused 50% (IC50) inhibition of cell growth. All experiments were carried out in triplicate wells and repeated three times^54–57^.

5 $$\mathrm{Relative\, cell\, toxicity} =\frac{\mathrm{Asample }-\mathrm{ Acontrol}}{\mathrm{Acontrol}}\times 100.$$

#### Annexin V/FITC staining for the apoptosis assay

To determine the effects of PTX-loaded nanocomposite, PTX void, and bare nanocomposite on apoptosis, WEHI-164 cells were stained with Annexin V-FITC/PI apoptosis detection kit (Sigma–Aldrich, USA) and analyzed by flow cytometry (B.D. Inc, USA). WEHI-164 cell lines were seeded in 6-well plates separately at a density of 105 cells per well and incubated to 85% confluence. Cells were treated with PTX-loaded nanocomposite, void PTX, and bare nanocomposite. After 48 h of incubation, cells were harvested by trypsinization, washed twice with cold PBS, and stained in binding buffer with 5 μl Annexin V/FITC for 5 min at RT, followed by 5 μl of P.I. reagent for 5 min at RT. The stained cells were placed on ice in darkness until analysis by flow cytometry.

#### RT-PCR

##### Isolation of total RNA and complementary DNA synthesis

Following a 48-h treatment period with WEHI-164 cells, total RNA was isolated from cell lysates using TRIzol (Invitrogen Life Technologies, UK). Optical density (OD) was measured at a wavelength of 260/280 nm to determine the concentrations and amount of RNA. Traditional cDNA synthesis kits required total RNA as an ingredient (Fermentas, Germany). As shown in Table 1, five different pairs of oligonucleotide primers were used, each of which was specific to an exogenous target or an endogenous gene. The instructions were provided by the kit manufacturer.

**Table 1:** Primers used for β-actin, Bcl-2, BAX, Bcl-xl, and Bak genes in the present study.

Primer name	Primer sequence Oligo sequence F (5′ → 3′)	Primer sequence Oligo sequence R (5′ → 3′)	References
β-Actin	CTGGCACCCAGCACAATG	GCCGATCCACACGGAGTACT	^58^
Bcl-2	TGCCTTTGTGGAACTGTACG	GGCCAAACTGAGCAGAGTC	^59^
BAX	AGCTGCAGAGGATGATTGC	GTTGAAGTTGCCGTCAGAAA	^60^
Bcl-xl	AAGGAGATGCAGGTATTGGTGAGT	CCAAGGCTCTAGGTGGTCATTC	^61^
Bak	ACTGGGATCGAGACATGTG	AGAAGGTGATGTGTACATTGC	^62^

##### Quantitative real-time polymerase chain reaction (PCR)

Real-time PCR was performed on an ABI prism to determine the levels of gene expression for β-actin, Bcl-2, BAX, Bcl-xl and Bak (Applied Biosystems, USA). β-Actin gene served as the reference control for this experiment. Each amplification process included 0.5 l of each specific primer, 5 l of cDNA, and 10 l of SYBR Green-I dye manufactured by Applied Biosystems (USA). PCR was carried out for 50 cycles, each cycle beginning at 95 °C for 10 min, 95 °C for 15 s, and 60 °C for 1 min. A melting curve (Table 2) was used to determine how well real-time PCR worked.

**Table 2:** Temperature, time, and the number of cycles for each step.

Step	Temperature (°C)	Time	Cycles
Initial denaturation	95	10 min	1
Denaturation	95	15 s	48
Annealing	60	1 min	
Melting curve analysis	95	5 s/step	1

### In vivo study

#### Animal use

The animal house provided us with female BALB/c mice that were between 5 and 6 weeks old and weighed 25 g each^63^. Animals were randomly divided into two test groups and two control group (n = 7 per group). Animals were housed under 12/12 light–dark cycle (light on at 6:00 a.m.) and the following laboratory conditions: temperature of 23 ± 2 °C and humidity of 55 ± 5%. Food and water were available ad libitum. All methods were performed in accordance with the ARRIVE guidelines. Guidelines for the care and use of laboratory animals served as our basis for the care and use of animals Al-Qasim Green University (ethics committee approval code: 533FD2). These guidelines were adopted by the Animal Care and Research Committee of Al-Qasim Green University. All in vivo methodologies and approaches were complied with the relevant norms and regulations. A committee from Al-Qasim Green University called the Animal Care and Research Committee, went over all experimental protocols, and gave their approval.

#### Hemolysis assay

A hemolysis experiment was used to study the release of hemoglobin from erythrocytes to evaluate the blood compatibility of SPION@Cs-PTX-PEG-FA, free PTX, and SPION@Cs-PEG-FA. This experiment was carried out to determine whether these three substances were compatible with human blood. The blood of the mice was collected in a separate 0.5 mm heparin tube, diluted 10 × with PBS, and centrifuged at 1800 revolutions per minute for 20 min. The concentration of acquired blood cells was increased to 2% (v/v) by first rinsing the precipitate with PBS and then centrifuging it at 1800 rpm for 20 min. Each sample solution included 10 µg of PTX, added with 200 µl of blood cells. After incubation of the combined suspensions at 37 °C for 4 h, they were centrifuged at 1800 revolutions per minute for 20 min. To calculate the amount of hemoglobin released, the absorbance of the supernatant produced was measured at 541 nm. In separate experiments, red blood cells were suspended in physiological saline and 0.2% Triton X-100 to achieve the desired levels of hemolysis of 0% and 100%, respectively.

#### Tumor-volume study and survival assay

The right flank of BALB/c mice received an injection of 1 × 10^6^ WEHI-164 cells suspended in 250 l of PBS. The injection was performed subcutaneously. The animals were divided into four groups, each with a total of seven individuals: Two test groups received 12.5 mg/kg BW (body weight) of SPION@Cs-PTX-PEG-FA and PTX removed intravenously for 3 weeks, and two control groups received SPION@Cs-PTX-PEG-FA and PBS. Both groups were given SPION@Cs-PTX-PEG-FA. The volume of tumors in cubic millimeters was measured three times a week using a digital Vernier caliper (Mitutoyo) until the mice were sacrificed on day 40 after injection. It was calculated using the formula length × width × height/6. Furthermore, the time that the mice survived was tracked as the primary criterion to establish whether the medication was effective^64^. We determined the median survival time, also called the percent IMST, as follows^65,66^:

6 $$\mathrm{IMST} =\frac{\mathrm{T}-\mathrm{C}}{\mathrm{C}}\times 100,$$

where T is the median survival time of the treated group, and C is that of the control group.

#### Splenocyte proliferation index

After completion of the therapy, each of the four groups of mice that had received the treatment was put to death. In the subsequent phase, the Baby et al. procedure^67^ was followed to remove the spleens of the animals. To extract a single-cell suspension from all spleens, they were first homogenized, filtered through a 100 µm membrane, and washed twice with PBS. To determine whether splenocytes were viable, density gradient centrifugation was performed on Ficoll/Hypaque and trypan blue (Sigma–Aldrich, USA) exclusion was used. In each well, 1 × 10^5^ splenocytes were planted in a total volume of 200 l of RPMI-1640 full medium. Cells were then mixed with tumor lysate and PHA (GIBCO) individually as positive and negative controls, respectively. The six-well plates containing the aforementioned cells were incubated for 5 days at 37 °C and a relative humidity of 5% CO_2_. MTT test was used to calculate the proliferation index of splenocytes. Each experiment was performed using three separate sets of wells.

#### ELISA study for measuring cytokines

The splenocytes were incubated with tumor lysate and PHA serving as a positive control. The medium served as the negative control at a density of 1106, which was based on an ELISA kit (R&D, Minneapolis, MN, USA) according to the manufacturer’ instructions. This step was carried out to estimate the amount of cytokines released when tumor lysate was added to the splenocytes.

### Statistical analyses

Statistical analyzes were performed with SPSS software version 24 and graphpad prism version 9. In both software programs, the statistical analysis was conducted by one-way ANOVA and unpaired students t-test. The significance level was established at *p < 0.05; *p < 0.01, and **p < 0.001.

### Ethics and permission to participate

This manuscript has not been previously released and is not now under consideration by any journal for publication.

## Result and discussion

### Effect of KMnO_4_ on SPIONs

**Figure 4:**
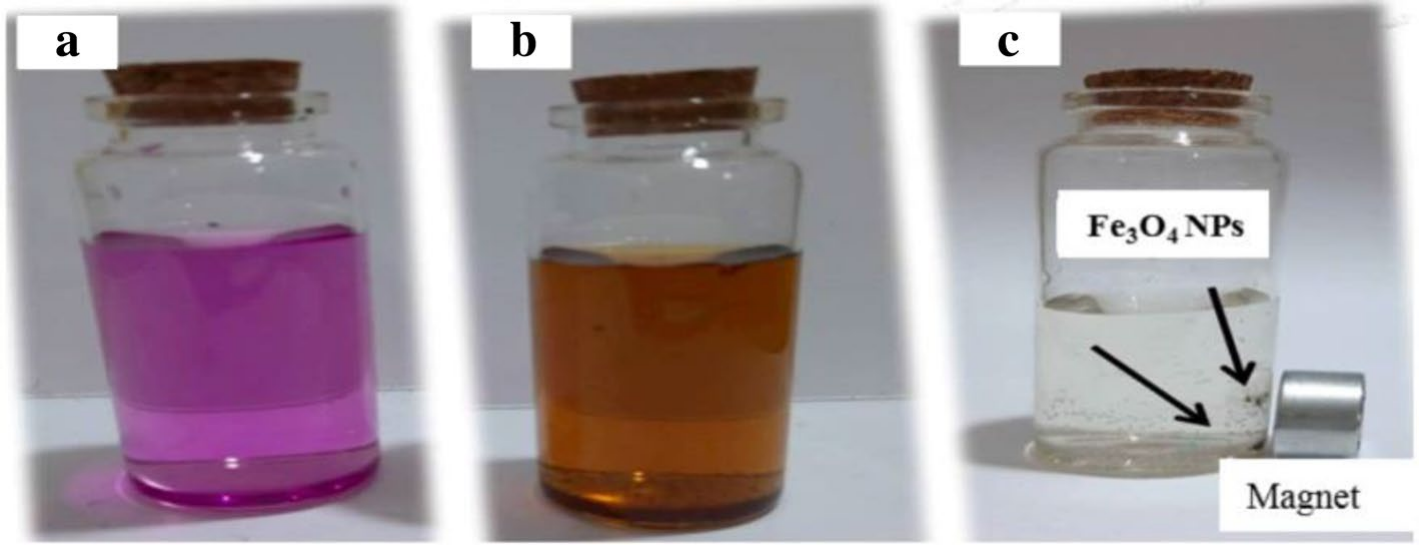
Color solution consists of DDW + KMnO_4_ before ablation (crimson black) in (**a**), color of Fe_3_O_4_ NPs colloidal solution after iron-plate ablation at a laser fluence of 28 J/cm^2^, in which the color is indicated in (**b**). The concentration (0.5 mg/10 mL) for Fe_3_O_4_ NPs formation in this solution is shown in (**c**).

The effect of KMnO_4_ as an additive in DDW was a strong oxidizing agent and can dissolve in water to give a dark crimson color solution, and its evaporation gives the crimson-black shown in Fig. 4a. Table 3 illustrates the advantages and properties, functions, and applications of permanganate potassium manganite. KMnO_4_ was used to determine iron (II) through its oxidation by KMnO_4_ to ferric iron used as a unique solution for nanocrystalline assembly and nanostructure fabrication interaction, as expressed by Eq. (1) ^19^.

**Table 3:** Some physical properties, functions, and applications of potassium permanganate.

	Properties	Functions	Applications	References
Permanganate potassium manganite	Inorganic compound with KMnO_4_ chemical formula	As a powerful oxidizing agent (E_o_ = 1.23 V)	Environmental (pollutants present in groundwater)	^13^
	Purplish-black color, crystalline salt, high activity, low cost, and nontoxic	- Catalyst in the energy-intensive process of potassium extraction - Used to make potassium hydroxide (KOH) salt	- Industrial applications - Biochemical footprint assays	^14^
	Dissolves in water as K^+^ and MnO_4_ and forms an intensely pink to a purple solution	- Used in the production of fertilizer - Used as a disinfectant and antiseptic agent	- Nanotechnology with permanganate oxidation - Dye adsorption and pseudo capacitance	^15^

The various mechanisms should ideally be examined independently, but this task was difficult given the extremely short time scales with the change in color of the solution indicated by the effect of wavelength in Fig. 4a (crimson black) before iron plate ablation. Together with the cavitation bubble generated, the expansion of the plume in the surrounding liquid causes a decrease in plasma temperature. The plasma acted as a reactor for the formation of NPs through the condensation of the atoms expelled from the metallic bulk (a bottom-up process) and depended on the amount of laser energy or wavelength. According to our findings, laser ablation combined top-down and bottom-up methodologies. The NPs produced by this method turned out to be a cubic inverse spinel structure packed in such a way that they were capable of exhibiting a structure either without a coating (referred to as "simple") or with a coating (core–shell)^67–69^. The latent heat of iron fusion was 13.8 kJ/mol, which may be the ablation threshold of the material. Figure 4b (golden) after ablation and Fig. 4c show the formation of Fe_3_O_4_ in solution, producing crystal structure inverse spinel magnetite NPs < 30 nm in diameter that exhibited superparamagnetic behavior^70,71^. This behavior indicated that in the absence of an external magnetic field, the particles had zero magnetization and less tendency to agglomerate^72–75^. Moreover, compared to metallic NPs, they had better chemical stability and compatibility with living things^8^. The NPs were stored at 4 °C in 100% isopropanol solution, and no visible oxidation was observed for less than a month^15^.

### UV–visible spectroscopy

**Figure 5:**
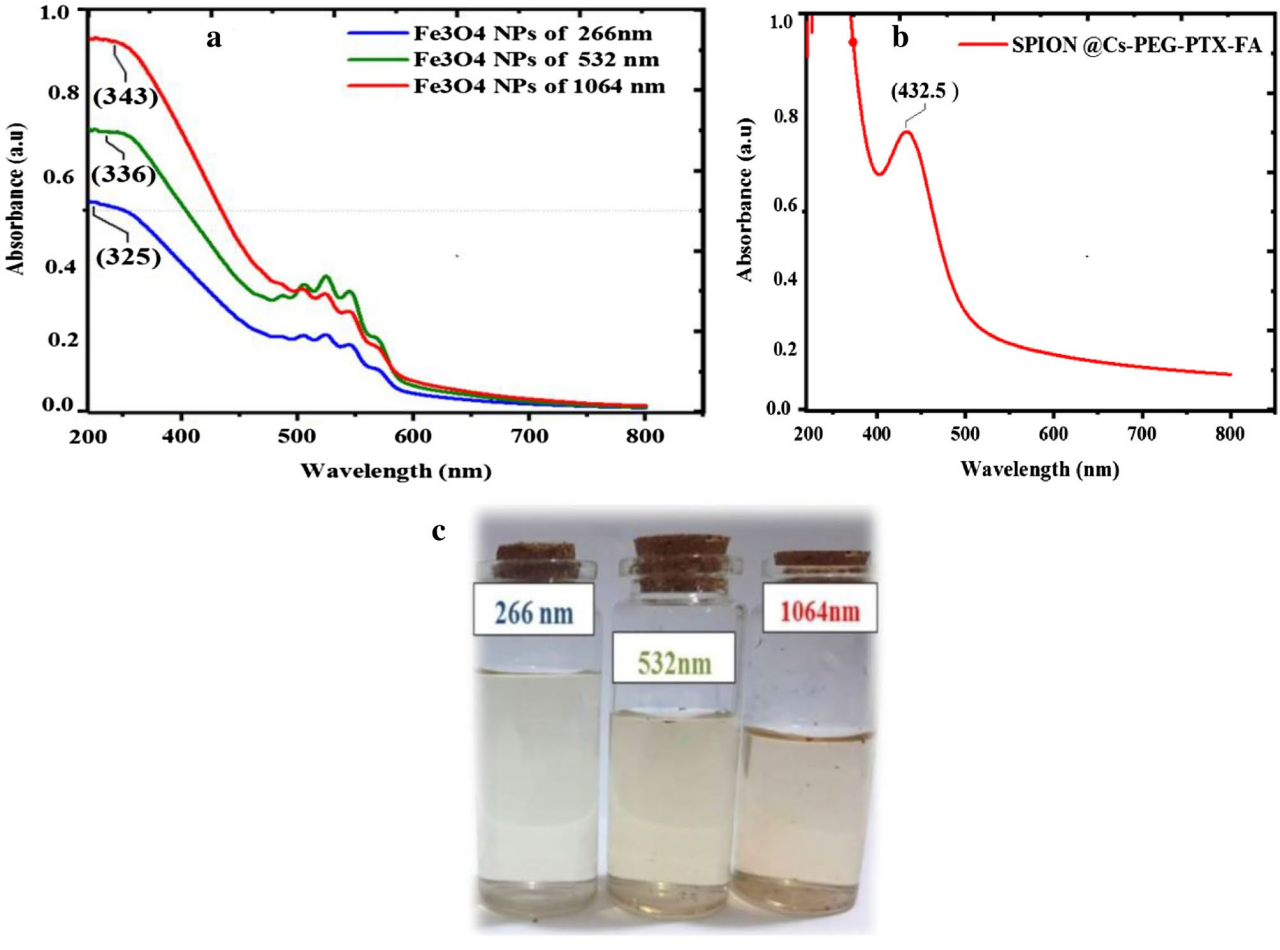
UV-Vis spectrophotometry absorption indicates a maximum absorbance above 300 nm at different wavelengths (1064, 532, and 266 nm) at a fluence of 28 J/cm^2^ in KMnO_4_ solution in (**a**); (**b**) shows the 100% absorption for SPION after coating by Cs + PEG + FA with the drug PTX; and (**c**) shows a photograph of the colloidal Fe_3_O_4_ NPs, in which the color indicates comparison between different wavelengths 266, 532, and 1064 nm.

The optical properties of colloidal NPs of Fe_3_O_4_ (in liquid phase) prepared at different wavelengths (266, 532, and 1064 nm) at a laser fluence of 28 J/cm^2^ and KMO_4_ solution conditions were investigated and measured. The particle distribution at 1064 nm was more homogeneous and had a high absorbance, the absorbance was > 0.9, but the Fe_3_O_4_ concentration at 266 nm was lower than that at 1064 nm. The absorbance at 532 and 266 nm at (0.7 and 0.5, respectively) is shown in Table 4 and Fig. 5a–c.

**Table 4:** Laser wavelengths and high peak shifts of SPION at 28 J/cm^2^ in KMnO_4_ solution.

Laser wavelength (nm)	High absorbance	Peak wavelength shifted (nm)
Fe_3_O_4_ NPs of 266	0.5	325
Fe_3_O_4_ NPs of 532	0.7	336
Fe_3_O_4_ NPs of 1064	> 0.9	343
SPION@ PTX-CS-PEG-FA	1	432.6

A general small shift towards long wavelength spectrum (red shift) and drop in absorbance occurred with decreased laser wavelengths, indicating that the amount of material that had been ablated was a lower quantity. This finding was also corroborated in a qualitative sense by the general reduction of color in the suspensions (insets in Fig. 5c). Although it was found that the spectra became smaller and decayed to zero as the experiment progressed accompanied by a tail oscillation that appeared in the region between 550 and 650 nm^76^. Due to the high reaction between DDW and KMnO_4_ that caused the instability of Fe_3_O_4_ NPs**,** we used zeta potential tests for Fe_3_O_4_ NPs before coating with polymers, and the results are shown in Fig. 11a,b.

### Structural analysis of nanocarriers

**Figure 6:**
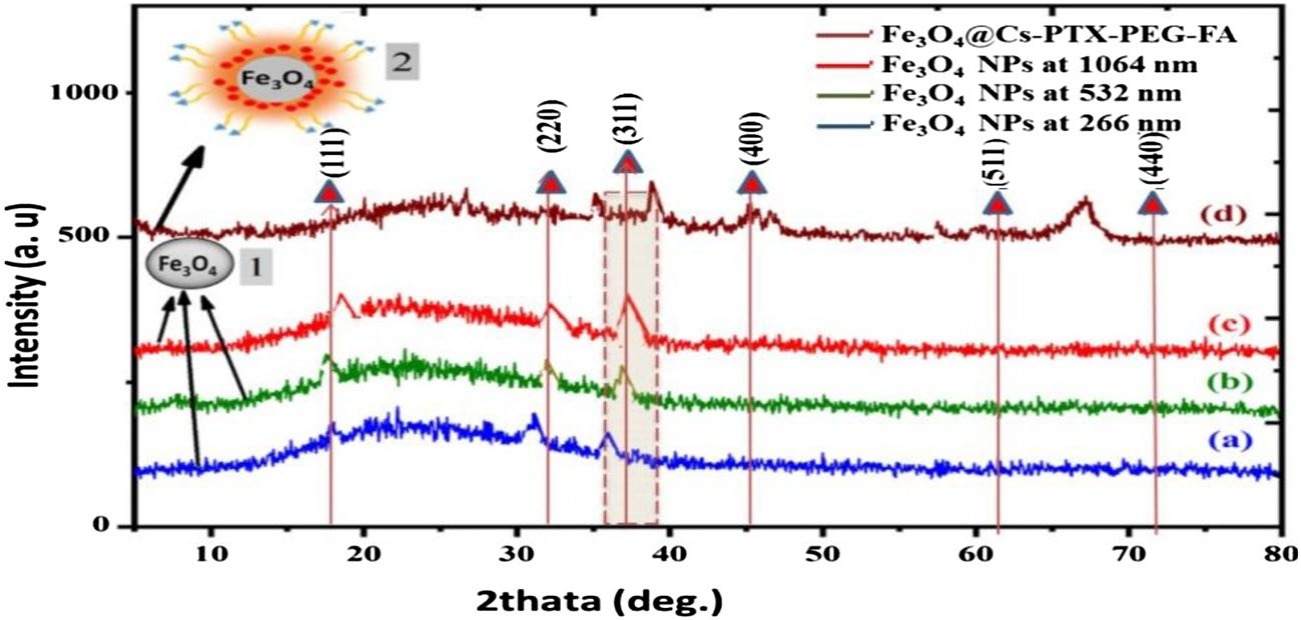
X-Ray diffraction patterns of Fe_3_O_4_ NPs (1) prepared by different wavelengths (1a, λ = 1064 nm; 1b, λ = 532 nm; 1c, λ = 266 nm in KMnO_4_ solution; and 2d, SPION@PTX-Cs-PEG-FA for λ = 266 nm).

The XRD patterns of laser-irradiated Fe plate in KMnO_4_ solution for varying wavelengths of 100 pulses with 266, 532, and 1064 nm and SPIONs after coating by polymers with drug PXT (SPION@PXT-Cs-PEG-FA) are shown in Fig. 6. The crystal structure of the sample was identified by XRD analysis.

The planes of reflection of Fe_3_O_4_ NPs at (a-266 nm, b-532 nm, c-1064 nm), and d-optimum synthesis of Fe_3_O_4_ NPs at wavelength 266 nm after polymer coating in Fe_3_O_4_ NPs (JCPDS file PDF no.65-3107) appeared at 2θ values of 18.52°, 30.29°, 35.54°, 45.30°, 57.20° and 67.18°, with the reflection plane having (hkl) values of (111), (220), (311), (400), (511) and (440), as shown in Fig. 6a–c. The low-intensity reflection planes for Fe_3_O_4_ NPs after laser ablation in KMnO_4_ solution and Fig. 6d high-intensity reflection planes for Fe_3_O_4_ NPs after coated SPION with polymers. The SPIONs had an inverse spinel structure without other phase-only magnetites with increased intensity and increased laser wavelength as reported by María et al.^84^. In an inverse spinel structure, eight Fe^3+^ ions occupy tetrahedral sites, while eight Fe^3+^ and Fe^2+^ ions randomly occupy octahedral sites and different intensities of peaks^77^. The XRD profile in Fig. 6d shows high-intensity peaks, indicating good crystallinity of the samples^71^ and that the wavelength of the laser influenced the growth of SPION nuclei. The grain size (D) of laser-irradiated SPION was calculated using peak broadening by Scherrer 's formula:

7 $${\text{D }} = \, 0.{9} \times \lambda \, / \, \left( {\beta \times {\text{cos}}\theta } \right),$$

where D is the average size of the crystallite, λ is 1.5406 Å, is the X-ray wavelength, β is the full width at half-maximum (FWHM), and θ is Bragg’s angle of diffraction. The mean size was determined from the FWHM of the XRD peak.

Quantitative analysis using the Debye–Scherer equation showed that the crystallite size of Fe_3_O_4_ NPs ranged from 7 to 35 nm depending on the laser wavelength under the same laser conditions as others Fig. 6a–d. All patterns. The samples also exhibited similar peaks with a slight change in position because the peak shifting to the lower (left shift for 266 nm) and higher (right shift for 1064 nm) angular positions also confirmed the presence of these tensile/compressive stresses compared to the stress-free peak positions. The development of residual stresses was directly related to the generation of lattice strains developed due to the implantation of plasma ions at interstitial sites and caused the presence of lattice defects and thermal shocks^78^. But the wavelength at 532 nm showed a peak between the two peaks of the long and short wavelengths, which is closer to the standard peak, and this means that this wavelength suffers little from crystallographic changes and internal stresses compared to other wavelengths, as shown in Fig. 6b.

Table 5 shows the various in max. Peak with the same lattice parameter for the Fe_3_O_4_ NP reflection plane with hkl (311), FWHM β, and grain size D irradiation in the KMnO_4_ solution. This shifting is due to changes in the surface energy that can compress the nanoparticle and, in this way, produce a shifting of the peaks to the right or generate a certain possibility that structural efforts be released.

**Table 5:** Variations in max. plane (311) maximum FWHM, D, and lattice parameters of Fe_3_O_4_ NPs after ablation with an Nd:YAG laser at different laser wavelengths and fluence of 28 J/cm^–2^ in KMnO_4_ solution.

Figure	Max. peak plane(hkl)	2θ (deg)	β = FWHM (deg)	D (nm) = Kλ/βcosθ	Shape
a, 266 nm	(311)	35.02	1.559	7.46017	Fe_3_O_4_ NPs (1)
b, 532 nm	(311)	35.647	1.427	6.87016	
c, 1064 nm	(311)	36.50	1.559	10.279	Fe_3_O_4_ NPs (1) SPION@PXT-Cs-PEG-FA (2)
d, (o.w) in 266 nm	(311)	36.601	1.18	99.9078	

However, the broadening of the peaks in XRD depended on the size of the crystallite, indicating that the crystallite size and residual macrostrains were interlocked with the broadening of the peak line and were difficult to separate from each other. Meanwhile, the composition was analyzed using XRD, and the results exhibited broad peaks with lower intensities, pointing to nano-sized domains rather than the larger domains observed in Fig. 6d. This finding was consistent with those of Hoang^71^. The sample that had larger particle sizes than the others displayed a peak with higher intensity probably due to the greater number of crystallographic planes present in that sample^78^.

### Characterization of magnetic nanoformulation

#### Morphology and size of SPION@Cs-PTX-PEG-FA

**Figure 7:**
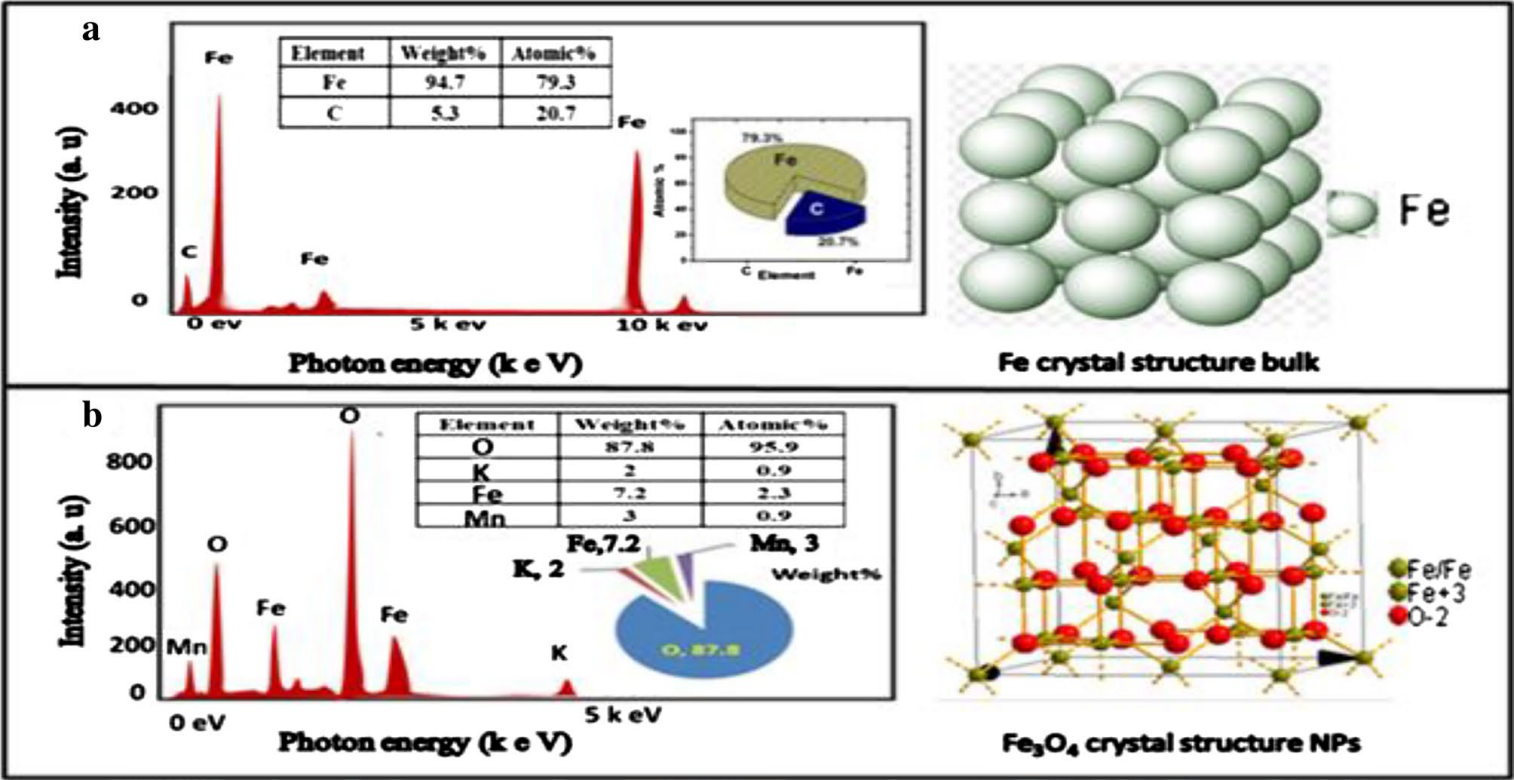
EDX of Fe-plate active material with crystal structure in (**a**), and EDX of SPION NPs in solution KMnO_4_ with a crystal spinel Fe_3_O_4_ NPs structure in (**b**).

The EDX spectrum and related analysis table of a sample are shown in Fig. 7a,b after being bombarded by an electron beam. The elemental composition of the NPs can then be determined. All elements were recognized using the K-series' distinctive X-ray before and after laser ablation target in liquid. It is a form of elemental analysis that is used to assess the chemical content of a material while microscopically examining it with our SEM equipment. The discrepancies were believed to be due to the short measurement period and interference from background and contaminant factors rather than the structural uniformity**.**

Figure 7a shows the strong peak of Fe before the pulsed laser ablation. However, this finding could be due to interference of their energy with the energies of iron and carbon or contamination from the background or SEM chamber. Figure 7b shows the results of EDX analysis of the sample prepared with 28 J/cm^2^, laser fluence for different wavelengths (266, 532, and 1064 nm). This figure also shows the strong peaks of Fe and O of the sample after ablation of an iron plate by pulsed laser in KMnO_4_ solution with weights and the atomic ratio of all elements. The lower ratio of Fe metal (79%) after ablation than before (0.9%) indicated the formation of iron oxide^15^.

#### Determination of the concentration of Fe_3_O_4_ NPs

**Figure 8:**
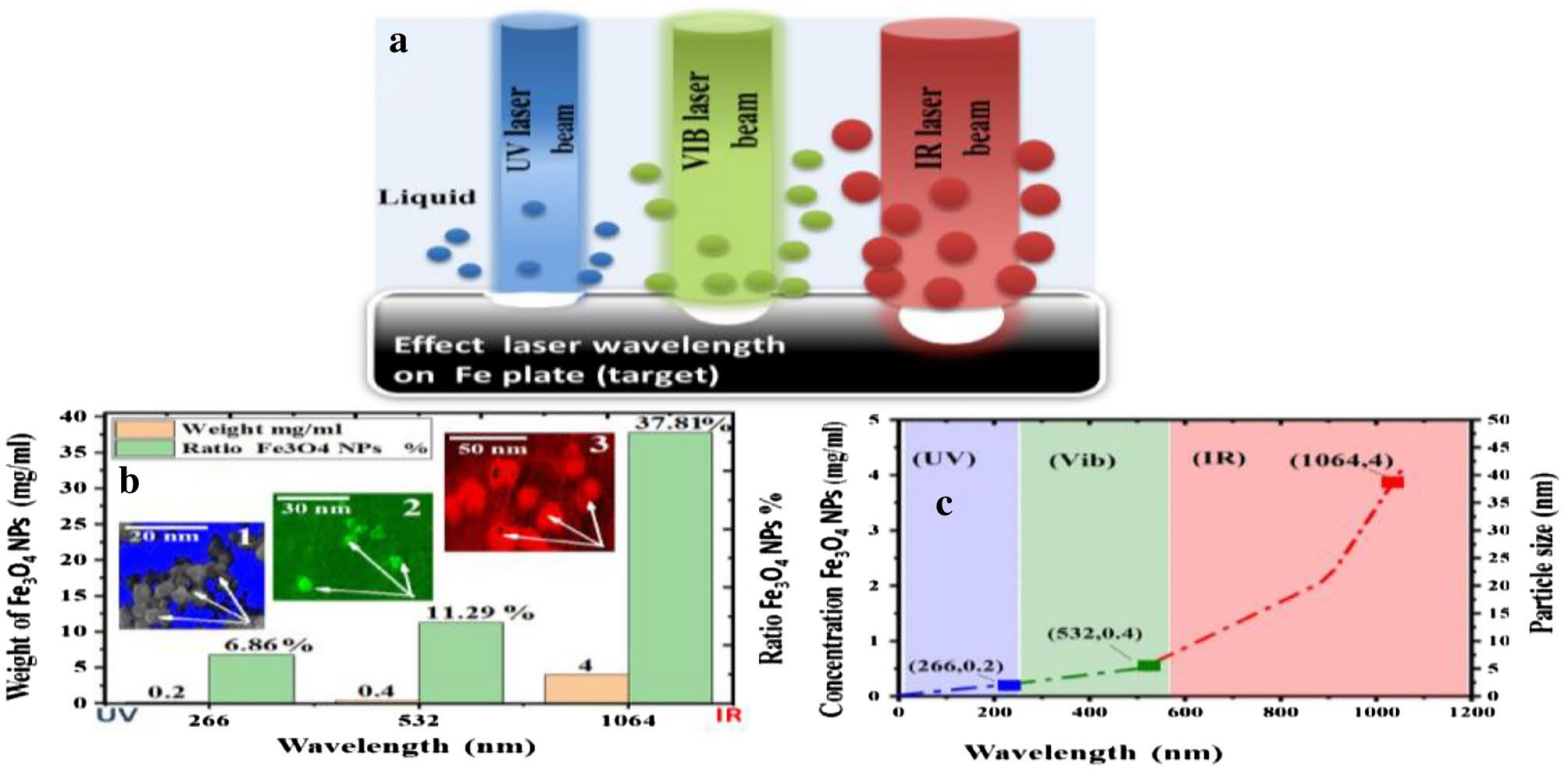
Schematic of the effect of different laser wavelengths (UV, Vib, and IR) on ablation of Fe plate in liquid by PLAL in (**a**), (**b**) charts showing the relationship between the weight of Fe_3_O_4_ NPs and wavelength indicating a maximum ratio of Fe_3_O_4_ NPs equal to 37.81% with 1064 nm and fluence of 28 J/cm^2^ in KMnO_4_ solution with images insert (1,2 and 3) as FESEM of SPION showing the size and shapes of NPs in all wavelengths; and (**c**) Shown fitted with concentration and different wavelengths (266, 532, and 1064 nm).

Figure 8a schematic of the absorption and preparation Fe_3_O_4_ NPs by different laser wavelengths UV (blue beam), Vis (green beam), and IR(red beam). This explains why a higher absorption cross section is given for Fe_3_O_4_ NPs of nearly Fe metal and a smaller spot size at UV &Vis wavelengths for laser beam compared to IR wavelengths. This is strongly indicated by the observed smaller NP diameters at shorter applied wavelengths, which may be the result of fragmentation induced by subsequent laser pulses during batch-PLA^79^.

Figure 8b charts the relationship between the maximum weight and the ratio of Fe_3_O_4_ NPs after laser ablation in KMnO_4_ solution at different wavelengths (266, 532, and 1064 nm). The results were in agreement with those of other analyses (UV, XRD, and FESEM images. In addition, Fig. 8b insert images (1, 2, and 3) indicate FESEM images shown in different sizes of Fe_3_O_4_ NPs due to different laser wavelengths^71^. This finding was due to a general decrease in concentration (pink bar) and Fe_3_O_4_ NPs ratio (green bar) at 0.22, 0. 4, and 4 mg/mL, as well as the Fe_3_O_4_ NPs ratio (green bar) at 11. 29%, 6.86%, and 37.81%, respectively. Thus, the number of nuclei was smaller and the particle sizes were larger at 1064 and 532 nm than at 266 nm in the KMnO_4_ solution^15^. The optimum values of Fe_3_O_4_ NPs concentration are taken in Table 6. Additionally, the Fig. 8c indicate the proportionality of concentration with different wavelengths (266, 532, and 1064 nm). This figure confirms that NPs sizes controlled and being under the influence of different wavelengths and each wavelength give certain NPs size.

**Table 6:** Optimal values of Fe_3_O_4_ NPs concentration in different wavelengths and liquid at a laser fluence of 28 J/cm^2^.

Laser wavelength (nm)	Weight (mg/ml)	Ratio of Fe_3_O_4_ NPs (%)
266	0.2	6.86
532	0.4	11.29
1064	4.5	37.81

Table 6 illustrates the optimal values of the Fe_3_O_4_ NPs concentrations in different laser wavelengths for determining the mass concentration of Fe_3_O_4_ NPs using a sensitive balance. We utilized four digits (0.0000) to calculate the amount of ablated material from the target (plate) by the laser ablation of the materials. For this purpose, the target was weighed before and after laser irradiation under all preparation conditions. After drying the targets, the amount of ablated target mass ΔM and the iron oxide decoration ratio were calculated from the concentration of Fe_3_O_4_ NPs. Therefore, through this process, notice the results of the highest concentration of Fe_3_O_4_ NPs, especially in these types of solutions with wavelength 1064 nm and then 532 and 266 nm at 0.4,0.04, and 0.02 mg/ml, respectively, at a laser fluence of 28 J/cm^2^ and.

#### TEM and electron diffraction (ED)

**Figure 9:**
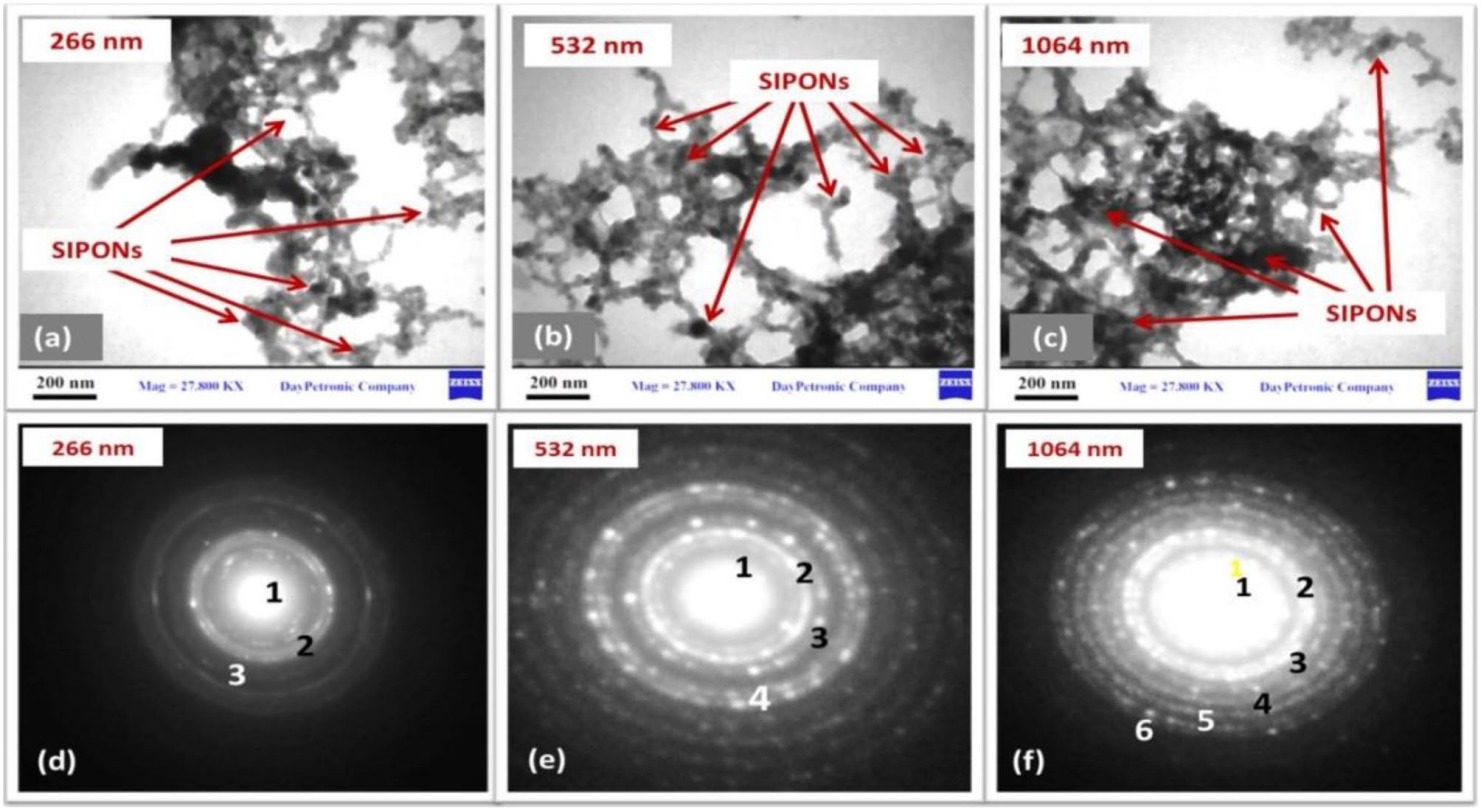
TEM images showing of Fe_3_O_4_ NP concentrations at different wavelengths: (**a**–**c**) 266, 532, and 1064 nm in KMnO_4_ solution. ED patterns of Fe_3_O_4_ NP (**d**–**f**).

The morphology of the obtained nanocomposites was investigated by TEM analysis at different wavelengths. Most of the particles were nearly cubic, with potentially good dispersity and minor agglomeration. The presence of agglomeration could be due to van der Waals forces for binding the particles together and also the shear forces that can be applied at the nanoscale. Figure 9a–c correspond to wavelengths of 266, 532, and 1064 nm, respectively. The Fe_3_O_4_ NPs aggregated because of the extremely high surface energy and magnetic dipole attractions among particles. The TEM image of Fe_3_O_4_ NPs in Fig. 9c shows a high concentration at a laser wavelength of 1064 nm. The TEM images also show that the average diameters of Fe_3_O_4_ NPs ranged from 5–10, 10–30 and 11–30 nm, respectively, as shown in Fig. 9, in agreement with the results calculated by the Debye–Scherrer formula^78^.

Coupled with electron diffraction results selected for the TEM area in Fig. 9d–f, we determined that the top portion was crystal. These patterns were obtained from TEM images of Fe_3_O_4_ NPs (SPIONs), and the results confirmed that the samples were crystal and had an inverse cubic spinel structure^71^. The diffraction rings were observed to be close to each other. The rings indexed in (111), (220), and (311) were observed to be close to the known lattice fringes of iron oxide in (1, 2, and 3), respectively, coated on the surface of iron oxide NPs. We also observed a homogeneous size and shape in all figures, but differences in Fe_3_O_4_ NP concentration resulted in more ring numbers in Fig. 9f at a laser wavelength of 1064 nm than in Fig. 9d, e due to the low density of SPIONs at laser wavelengths 532 and 266 nm.

**Figure 10:**
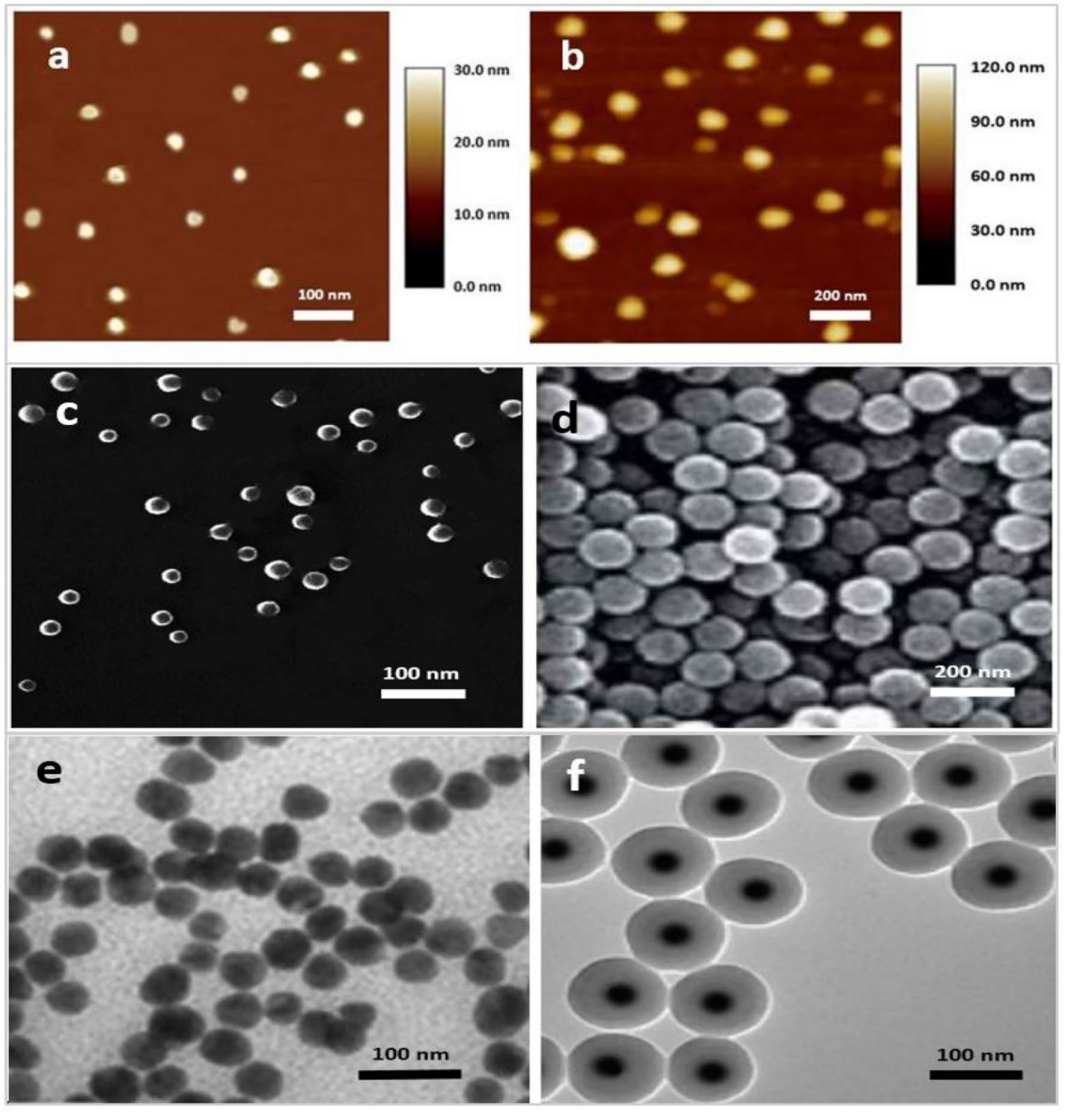
Characterization of SPIONs and SPION@Cs-PTX-PEG-FA nanoformulation with different techniques. (**a**), (**c**) and (**e**) are related to atomic force microscopy, scanning electron microscopy, and transmission electron microscopy images of SPION respectively and the (**b**), (**d**) and (**f**) are related to atomic force microscopy, scanning electron microscopy, and transmission electron microscopy images of SPION@Cs-PTX-PEG-FA respectively.

**Figure 11:**
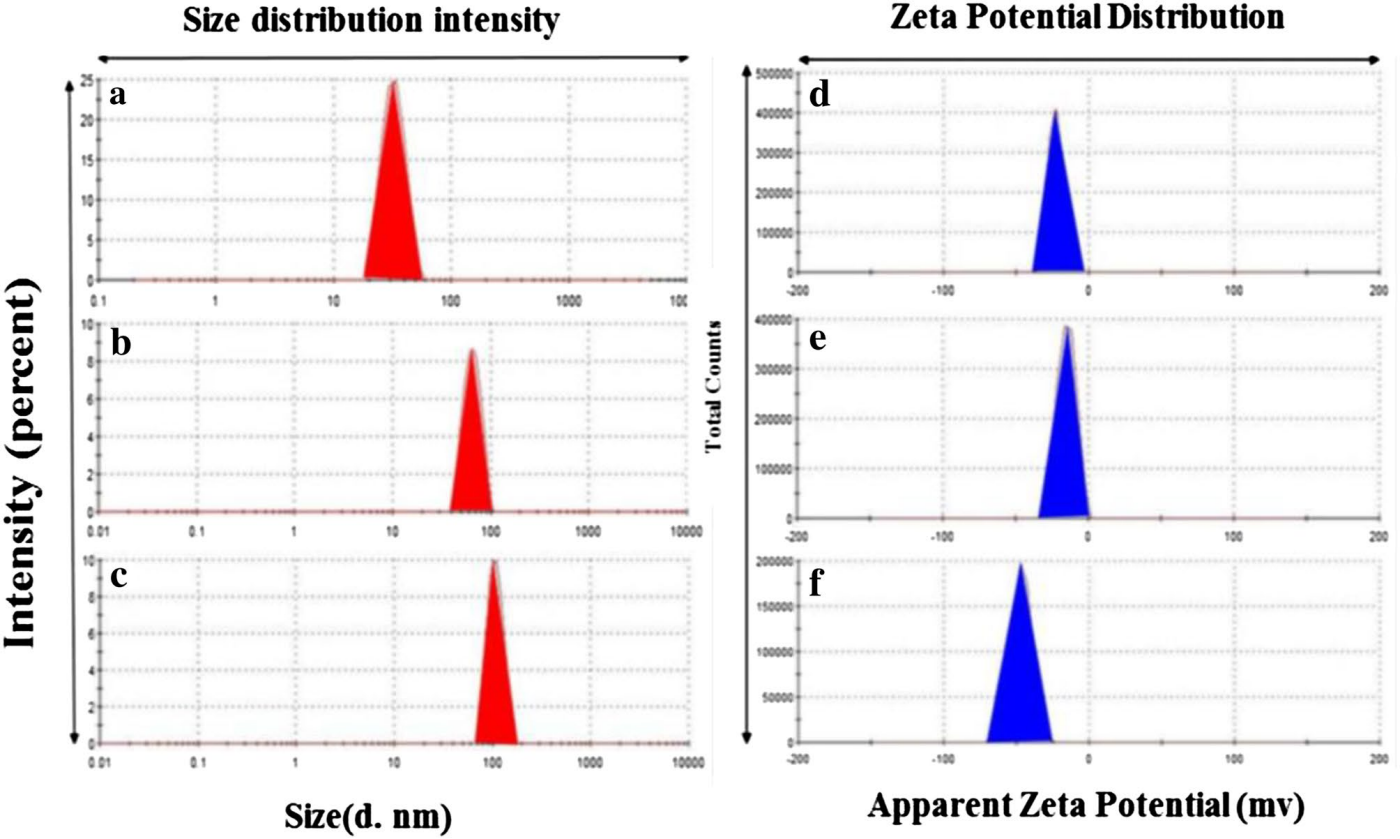
In the right illustrated: Particle-size distribution of (**a**) SPIONs, (**b**) SPION@Cs-PTX, and (**c**) SPION@Cs-PTX-PEG-FA. In the left illustrated: Zeta-potential charge measurement of (**d**) SPION, (**e**) SPION@Cs-PTX, and (**f**) SPION@Cs-PTX-PEG-FA obtained by dynamic light scattering.

After determination of encapsulation efficiency and loading content of PTX that were 84.7 ± 0.19% and 15.28 ± 0.5% respectively, the morphological studies of the prepared of Fe_3_O_4_ NP and SPION@Cs-PTX-PEG-FA using AFM (Fig. 10a,b), SEM (Fig. 10c,d), and TEM (Fig. 10e,f) indicated their spherical shape, stability, polydispersity, and uniform size. The particle size and its distribution were measured by dynamic light scattering (DLS; Malvern Zetasizer ZS, Malvern, UK). The SPION@Cs-PTX-PEG-FA exhibited suitable physical stability, spherical shape, desirable size (~ 102.6 nm), negative zeta-potential charge (− 47.5 mV), and suitable polydispersity (~ 0.047 mV) (Fig. 11). The size of the particles is one of the most important criteria that can be controlled to influence the biocompatibility and bioactivity of NPs. Particles size was another important consideration because of its close connection to the formulation of a stable nanocarrier^80,81^. In this work, the synthesized SPION indicated 31.89 ± 6.9 nm average diameter size of 31.89 6.9 nm. This diameter size range is suitable for sequence coating processes because the final average particle size should be suitable for drug delivery purposes. Indeed, 100 nm nanoparticles had a 2.5-fold greater uptake rate than 1 μM microparticles, and a sixfold greater uptake than 10 μM microparticles. However, particles 200 nm are not very pursued, and nanomedicine often refers to devices 200 nm (i.e., the width of microcapillaries). Coating the SPION with Cs polymer and then PTX drug loading of SPION@Cs caused to shift the diameter size from 31.89 ± 6.9 nm (Fig. 11a) to 63.3 ± 12.55 nm (Fig. 11b). Here, the main reason for the increase in size is the addition of the Cs polymer because the polymer contains the drug in its tissue as dissolved molecules with low molecular weight. In comparison, the entire drug-loaded SPION@Cs-PEG-FA (102.6 ± 17.66) was approximately 40 nm larger than its SPION@Cs-PTX form (Fig. 11c). This was due to the coating of SPION@Cs-PTX with PEG and FA, respectively. Furthermore, the high level of negative charge (− 47.5 ± 7.36 mV) leads to a suitable polydispersity ratio (~ 0.047 mV) due to the remarkable repulsion between NP. More precisely, SPION exhibited a negative zeta potential (ζ) of − 14.3 mV (Fig. 11d). This result It was largely consistent with the results of Magda et al.^82^. They achieved the − 21.8 ± 5.8 mV zeta potentials for Fe_3_O_4_ NPs. The negative zeta potential of SPION is due to negative charge of its components (O^2−^). SPION coated with Cs and PTX had a different zeta potential of − 14 ± 6.16 Mv (Table 7). After the SPION coating process with Cs and PTX, the zeta potential changes to less negativity in nanoparticle charge (Fig. 11e). Therefore, this Zeta potential measurement indicates the successful coating of SPION. The decrease in the negative charge density of the SPION@Cs-PTX is attributed to the presence of the NH_3_ group of Cs. On the other hand, the positive charge of PTX, helps decrease the negative zeta potential level of PTX loaded SPION@Cs nanoformulation. Ahmed et al. indicated that increasing the PTX ratio in conjugate with chitosan will increase the zeta potential to more positivity of the nanostructure^61,88–94^. An additional coating process with PEG and FA presented a high shifting value to negative zeta potential. This was due to the negative charge of both two of these molecules. The PEG molecules possess negative nature due to the presence of high levels of (OH^−^) groups in their structure. However, the negligible negative physiological charge of FA also aids in shifting the entire SPION@Cs-PTX-PEG-FA nanoformulation to a negative zeta potential of (− 47.5 ± 7.36 Mv) (Fig. 11f). Finally, due to the fact that the zeta potential charge between ± 40 and ± 60 is classified in the appropriate range^23^, the nanoparticle obtained appears to be very suitable in terms of stability. This zeta potential level (− 47.5 ± 7.36 mV) leads to repulsion between nanoparticles due to the charge on their surfaces and inhibits the aggregation events of SPION@Cs-PTX-PEG-FA nanoformulations.

**Table 7:** Particle size and surface charge of SPIONs, SPION@Cs-PTX, and SPION@Cs-PTX-PEG-FA.

NP type	Particle size (nm)	Zeta potential (mV)	PDI
SPION	31.89 ± 6.9	− 21.8 ± 5.8	0.002
SPION@ Cs-PTX	63.3 ± 12.55	− 14 ± 6.16	0.041
SPION@Cs-PTX-PEG-FA	102.6 ± 17.66	− 47.5 ± 7.36	0.047

**Figure 12:**
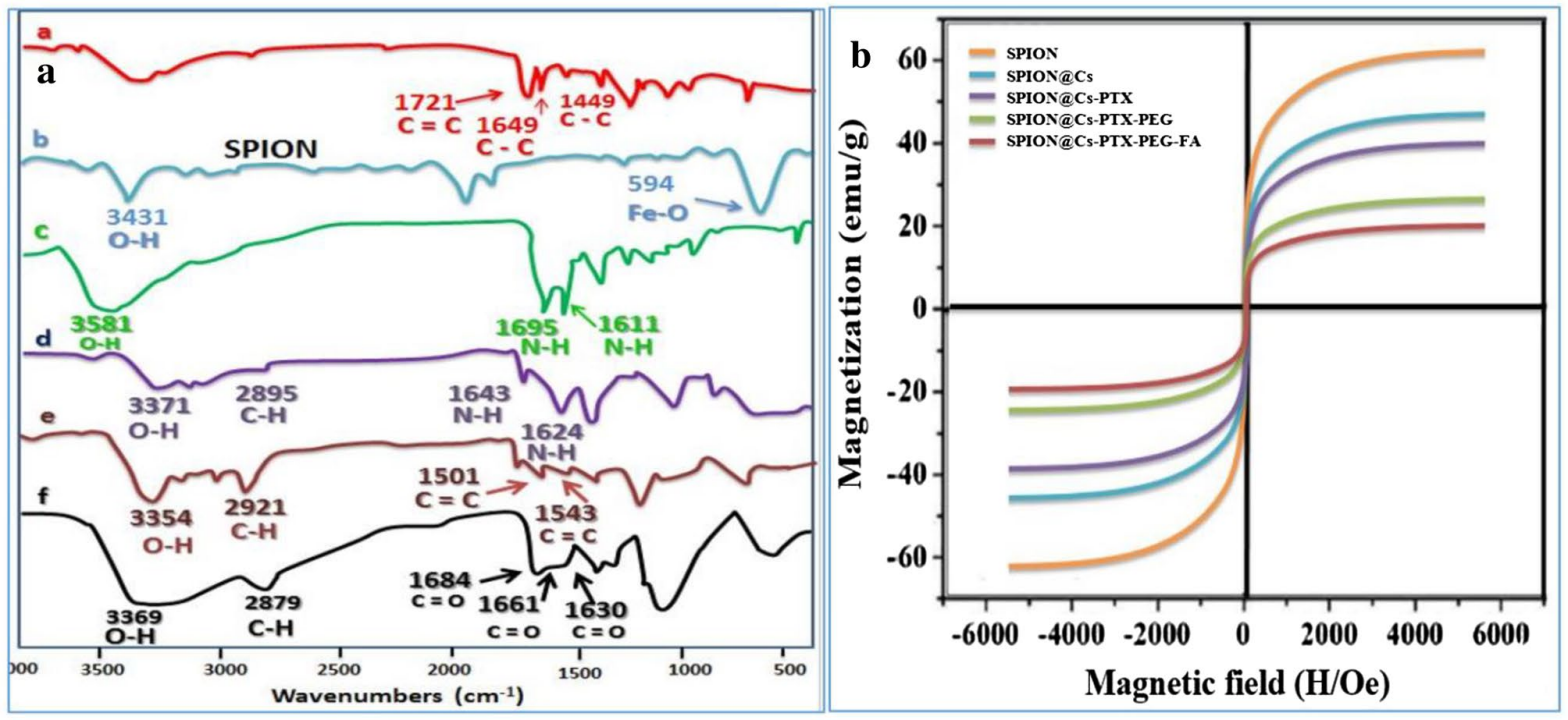
(**a**) FTIR spectra of PTX (a), SPION (b), FA (c), SPION@Cs (d), SPION@Cs-PTX-PEG (e), and SPION@Cs-PTX-PEG-FA (f). (**b**) VSM of different formulations of SPION.

The FTIR spectra of SPION, SPION@Cs, SPION@Cs-PEG-FA, and PTX-loaded SPION@Cs-PEG-FA are shown in Fig. 12. In the SPION spectra, the absorption peak at 592 cm^−1^ was characteristic of Fe–O–Fe in SPIONs. However, these characteristic Fe–O–Fe peaks transferred to 587, 563, and 554 cm^−1^ for SPION@Cs, SPION@Cs-PEG-FA, and PTX-loaded SPION@Cs-PEG-FA, respectively. Compared with bare SPION, the spectrum of Cs-SPION had two characteristic peaks of Cs at 1634 and 1068 cm^−1^ owing to the C–O that was linked with the –NH_2_ group and glycosidic bond stretching vibrations, respectively. In the SPION@Cs-PEG-FA spectrum, the maximum strength at 1098 cm^−1^ increased due to the C–O–C stretching vibration of the PEG structure. The new intensive peaks at 1703 cm^−1^ were due to the amido link between PEG and FA, whereas that at 1613 cm^−1^ can be attributed to the benzene ring of FA. In the spectra of PTX-loaded SPION@Cs-PEG-FA, some additional peaks were detected at 824 cm^−1^ (corresponding with the stretching bands of C–O–CH_3_ from PTX), whereas those at ∼ 865 and 775 cm^−1^ corresponded with the primary amine NH_2_ wag and N–H deformation bonds from PTX, respectively.

Furthermore, the spectrum of SPION-Cs-PTX-PEG-FA had two peaks at 1601 and 1543 cm^–1^ for the aromatic C–C bond. These results further indicated that the surfaces of naked SPION were coated with a shell of Cs and subsequently the Cs was decorated with FA-PEG conjugates. Furthermore, a characteristic peak at 1684 cm^–1^ of FA also appeared in the FA-functionalized SPION-Cs-PTX-PEG peak, indicating that FA was conjugated to PEG. The conjugation of FA-PEG and PEG onto the Cs-coated SPION surface was characterized by the new amide bond (CONH). Comparing the spectrum of Cs-coated SPION NPs, SPION-Cs-PTX-PEG NPs, and FA-functionalized SPION-Cs-PTX-PEG NPs revealed two new sharp peaks at 1661 and 1630 cm^−1^ corresponding to the bonded C=O groups of the amide bond for SPION-Cs-PTX-PEG NPs and FA-functionalized SPION-Cs-PTX-PEG, respectively. VSM was used to evaluate the magnetic capacity of the SPION-Cs-PTX-PEG-FA nanoformulation (Fig. 12b). These results proved the superparamagnetic properties of SPION NPs with a saturation magnetization value of 59.6 emu/gm. The curves also presented the superparamagnetic properties of the SPION-Cs-PTX-PEG-FA nanosystem with a saturation magnetization value of 19.7 emu/gm. This experiment confirmed that the fast response of all to the external magnetic field and stable structure of SPION-Cs-PTX-PEG-FA in solution make these NPs suitable nanocarriers.

### In vitro studies

#### Cell internalization

**Figure 13:**
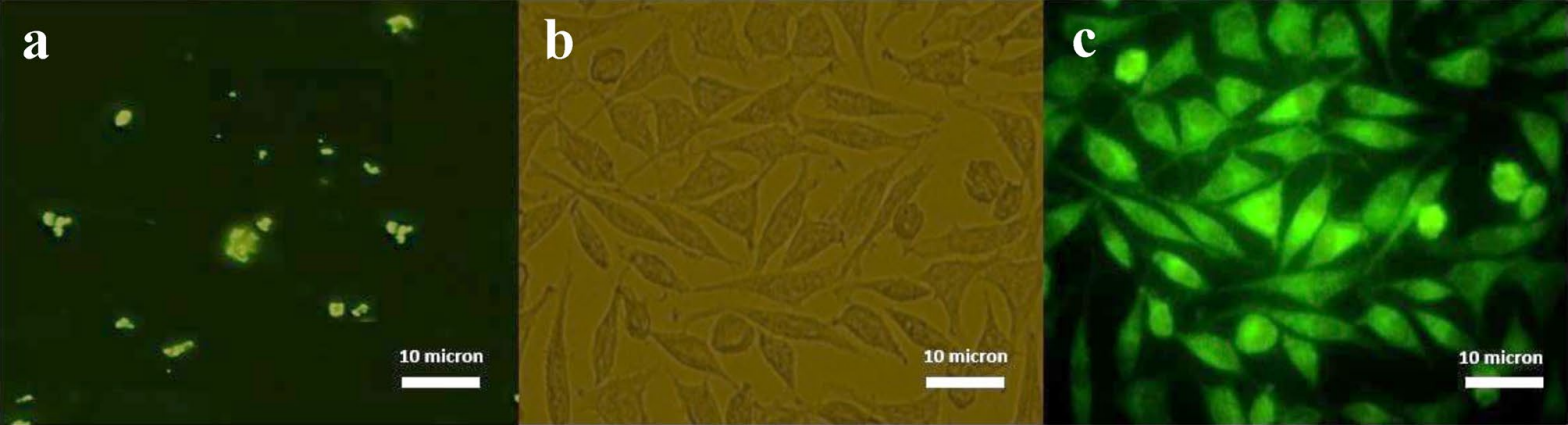
Evaluation of FITC-SPION-Cs-PTX-PEG-FA cell uptake by fluorescence imaging in WEHI-164 cancer cells (×400 magnification). Image of cells treated with PTX with fluorescence microscopy (**a**). FITC-SPION-Cs-PTX-PEG-FA-treated cells using optical microscopy image (**b**). Fluorescence imaging of cells treated with FITC-SPION-Cs-PTX-PEG-FA (**c**).

Stimulation of PTX uptake in WEHI-164 cancer cells and by its decorated by FITC, performed by fluorescence imaging. As shown in Fig. 13, the treated cells with FITC decorated nano-drug showed green due to FITC-SPION-Cs-PTX-PEG-FA uptake due to the solubility enhancement of PTX. In contrast, the void PTX-treated cells illustrate green and star-like sedimentations in intercellular space due to PTX water insolubility**.**

#### MTT assay

**Figure 14:**
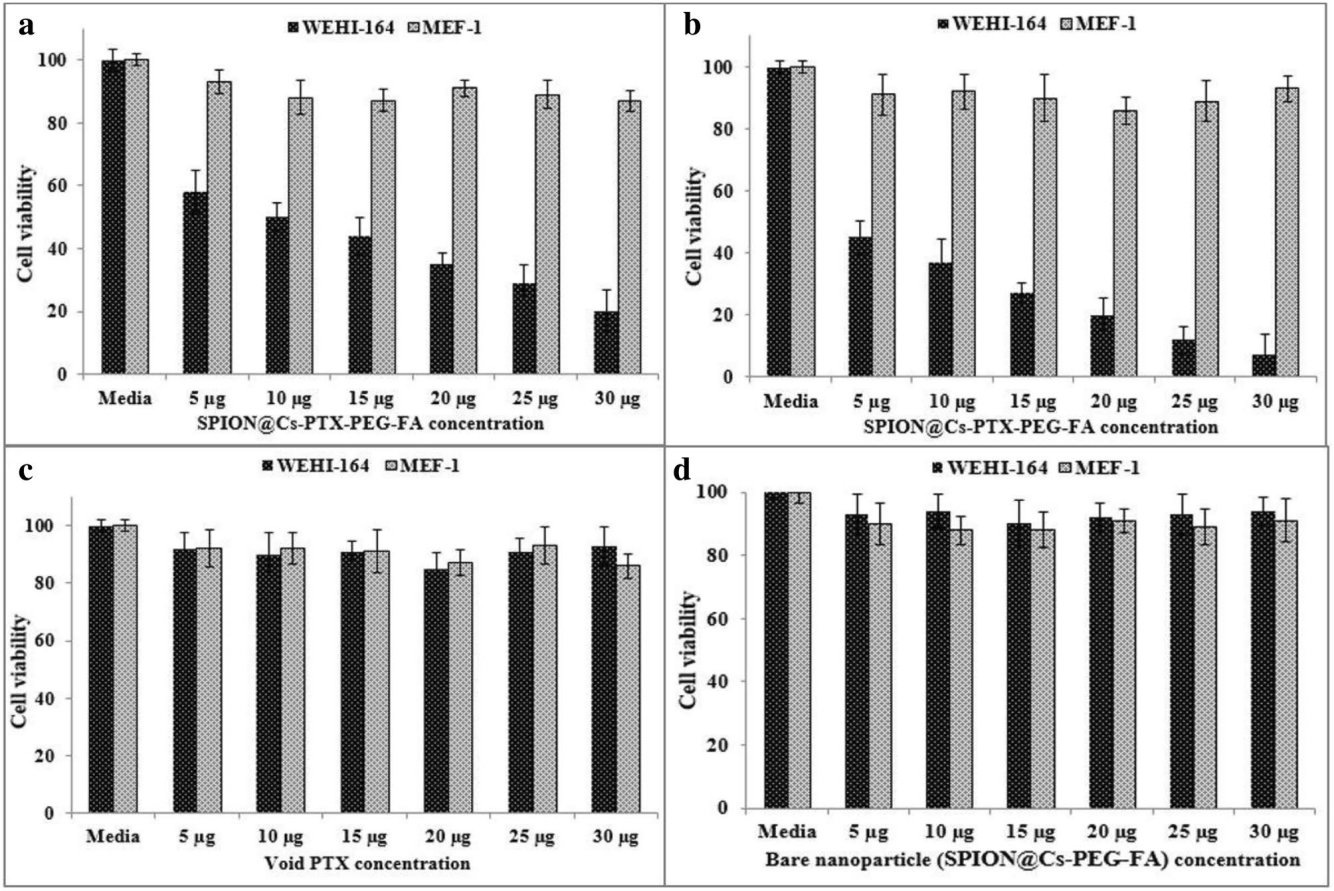
Cell-toxicity evaluation of SPION@Cs-PTX-PEG-FA nanoformulation by MTT assay on WEHI-164 and MEF-1 cell lines with 24 h (**a**) and 48 h (**b**) incubation. The cells were also treated with void PTX (**c**) and bare SPION@Cs-PEG-FA NP (**d**) at the same concentration for 48 h (mean ± SD (*p < 0.05; **p < 0.01; ***p < 0.001); n = 3).

Figure 14 shows the cytotoxicity findings of PTX-loaded SPION@Cs-PEG-FA investigated by the MTT test against the WEHI-164 and MEF-1 cell lines. Undiluted PTX and undiluted SPION@Cs-PEG-FA were used to eradicate the cancer cells. Even at the highest concentration of 30 µg, no evidence of cell toxicity was found. Furthermore, more than 90% of the cells were still alive after 48 h of incubation, demonstrating that they were cytocompatible. When cells were treated with PTX-loaded SPION@Cs-PEG-FA, the vitality of the cells decreased dramatically, suggesting a higher level of inhibitory activity of cancer cells compared to free PTX and bare SPION@Cs-PEG-FA alone. In contrast, normal cells showed almost no sign of cell toxicity in response to any of the treatments, even at the maximum concentration used. The dose–response curve fitted to the cell-viability data allowed for the determination of IC_50_ concentration. Within 24 and 48 h, the IC_50_ values of SPION@ Cs-PEG-FA for the WEHI-164 cancer cell line were 10.34 and 6.72 g, respectively. Mohammad et al. indicated that PTX nanoformulation causes a seven-fold reduction in IC_50_ and an eight- to nine-fold decrease in dose compared to free PTX^83^. Du et al. further evaluated the in vitro cytotoxicity of murine melanoma cells by MTT test. The cells were B16 F10. The IC_50_ value of PTX was found to be 293.8 12.23 and 21.85 18.33 nM after 36 and 48 h, respectively. This value was 3.4-fold greater than that of PTX/NCs, indicating that PTX/NCs were more effective in killing cancer cells^84^.

#### Apoptosis assays

**Figure 15:**
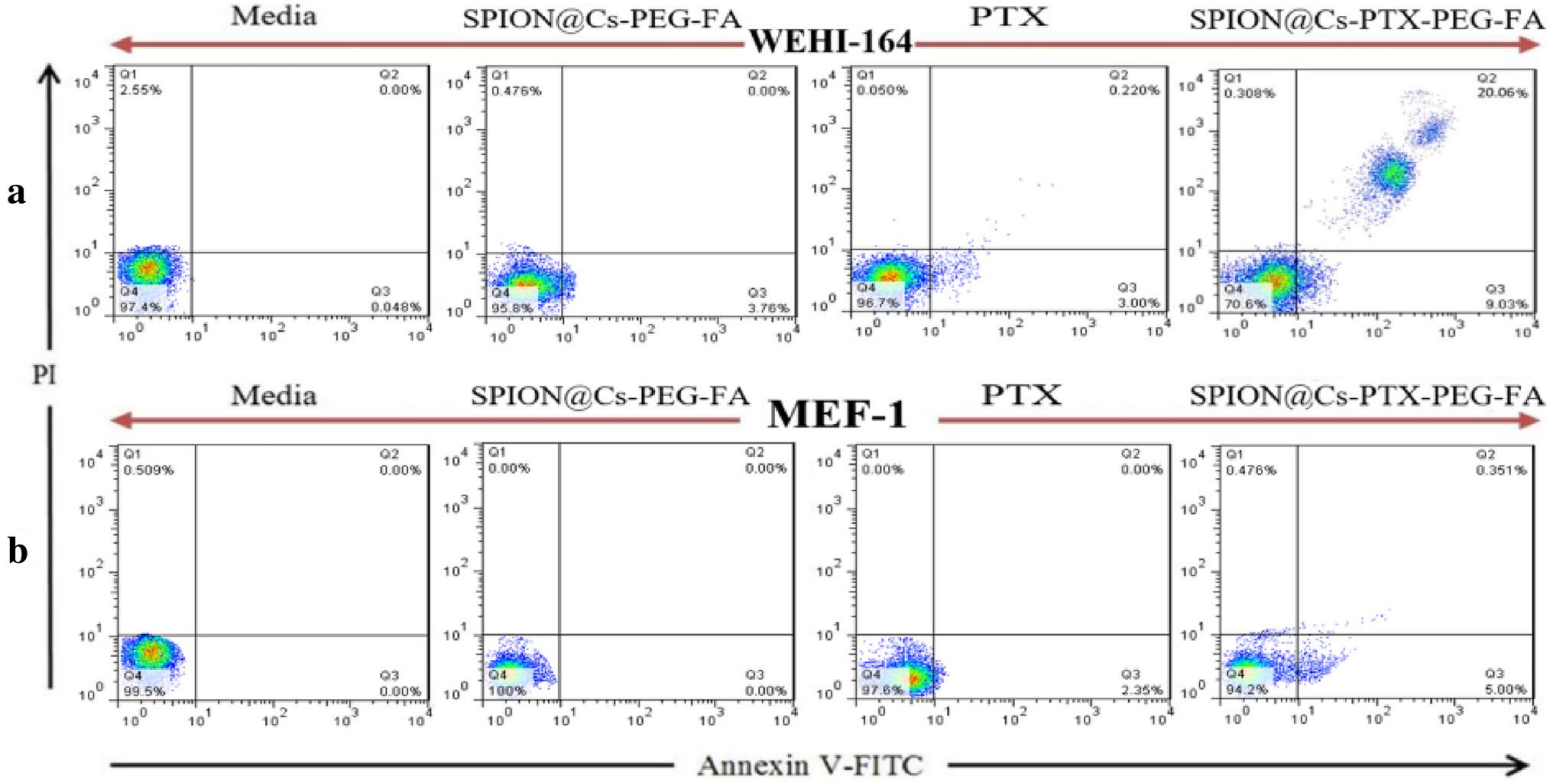
Apoptosis induction of WEHI-164 (**a**) and MEF-1 (**b**) cell lines with media (control), bare SPION@Cs-PEG-FA NPs, free PTX, and SPION@Cs-PTX-PEG-FA. The number of WEHI-164 cells that undergo apoptosis increased significantly when treated with SPION@Cs-PTX-PEG-FA. Treatment of the WEHI-164 cell line with bare SPION@Cs-PEG-FA and free PTX separately indicated that both treatments did not show any notable induction of apoptosis. Treatment of the MEF-1 cell line with bare SPION@Cs-PEG-FA NP, free PTX, and SPION@Cs-PTX-PEG-FA separately proved that all treatments did not show any noticeable induction of apoptosis.

The ability of the PTX-loaded nanocomposite SPION@Cs-PTX-PEG-FA to induce apoptosis was subsequently evaluated through flow cytometry by Annexin V-FITC/PI dual staining using WEHI-164 and cells treated with either free PTX or bare nanocomposite (SPION@Cs-PEG-FA). Live cell population, early apoptosis, late apoptosis, and necrosis were represented as the percentage of Annexin V-FITC/PI, Annexin V-FITC^+^/PI and Annexin V-FITC/PI^+^ and Annexin V/FITC^+^/PI^+^, respectively. SPION@Cs-PTX-PEG-FA significantly stimulated more apoptosis as revealed by flow cytometry analysis using Annexin V-FITC/PI staining. The percentage of apoptotic cells was approximately 75% and 73% in FA-PEG-PTX-Cs-SPION compared to approximately 43% and 39% in free PTX for WEHI-164 cell lines, respectively. We also observed that the SPION@Cs-PEG-FA system exhibited a negligible amount of apoptotic and necrotic cells with up to 95% live cells for WEHI-164 cell lines, proving that SPION@Cs-PEG-FA was biocompatible at the tested concentrations. A higher percentage of apoptotic or necrotic cells, when treated with SPION@Cs-PTX-PEG-FA, could be explained by the higher uptake of nanoformulation owing to the targeting ability of FA to FA receptors in cancer cell lines followed by efficient release of PTX (Fig. 15). The results of the apoptosis assay were in agreement with the high anticancer activity of PTX shown in the cytotoxicity assay and the high cellular uptake of SPION@Cs-PTX-PEG-FA. Apoptosis was triggered in tumor cells by the PTX protein, which worked by inhibiting cell division^85^ and stabilizing the microtubule assembly through non-covalent interactions with the cytoskeleton. Wang et al. demonstrated that the PTX nanoplatform manufactured can successfully trigger apoptosis and autophagy in cells^86^. The fabricated PTX-loaded SPION also demonstrated inhibition of the targeted cell proliferation and migration, as well as programmed cell death. This programmed cell death occurred through both apoptosis modulated by a burst of ROS and autophagy, with an accumulation of autophagosomes and LC3-II signals detected in treated glioblastoma U251 cells after uptaking^85^.

#### Real-time PCR and gene expression analysis

**Figure 16:**
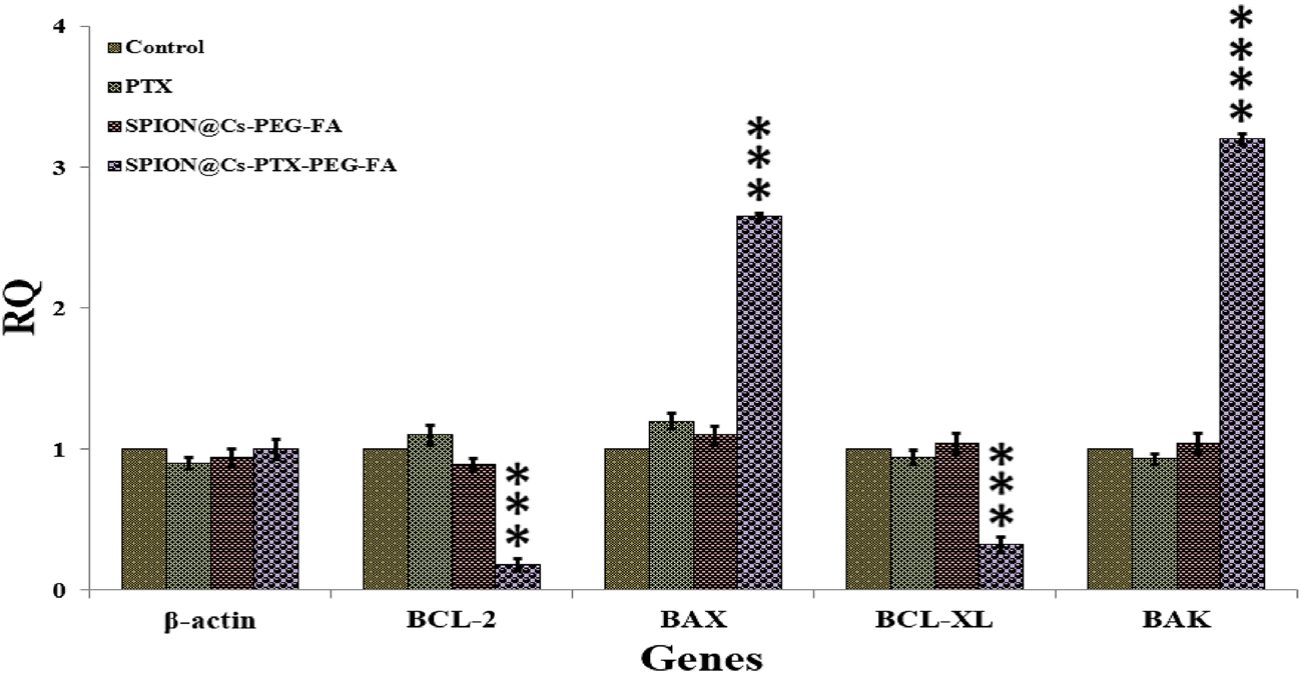
Gene expression of BCL-2, BAX, BCL-XL, and BAK analyzed in four groups of WEHI-164 cells. In each group, the first column represents control cells, the second represents cells treated with PTX, the third represents cells treated with SPION@Cs-PEG-FA, and the fourth represents cells treated with SPION@Cs-PTX-PEG-FA. The values in the graph indicate the mean ± SD. ***p < 0.001, ****p < 0.0001 indicate significant differences between the control (untreated) and other treatments.

We analyzed the expression levels of the genes BCL-2, BAX, BCL-XL, and BAK to better understand the mechanism (or pathways) that mediated the apoptosis triggered by SPION@Cs-PTX-PEG-FA. As mentioned above, quantitative PCR was used to investigate the expression levels of the BCL-2, BAX, BCL-XL, and BAK genes. β-Actin gene served as the reference control gene (housekeeping gene). As shown in Fig. 16, the expression levels of each candidate gene differed substantially in the malignant samples compared to the non-malignant ones. Whether they were treated with PTX did not lead to a discernible difference in the amount of β-actin expression between the control and malignant cells. However, BAX and BAK expression was significantly higher in cancer cells treated with SPION@Cs-PTX-PEG-FA compared to PTX void and bare SPION@Cs-PEG-FA (****p 0.0001) (Fig. 16). Previous findings about the same drug in nanocarrier forms showed the same result in the expression levels of pivotal genes related to apoptosis (ancogenes and proto-ancogenes)^87^.

### Evaluation of in vivo antitumor efficiency

#### Hemolysis

**Figure 17:**
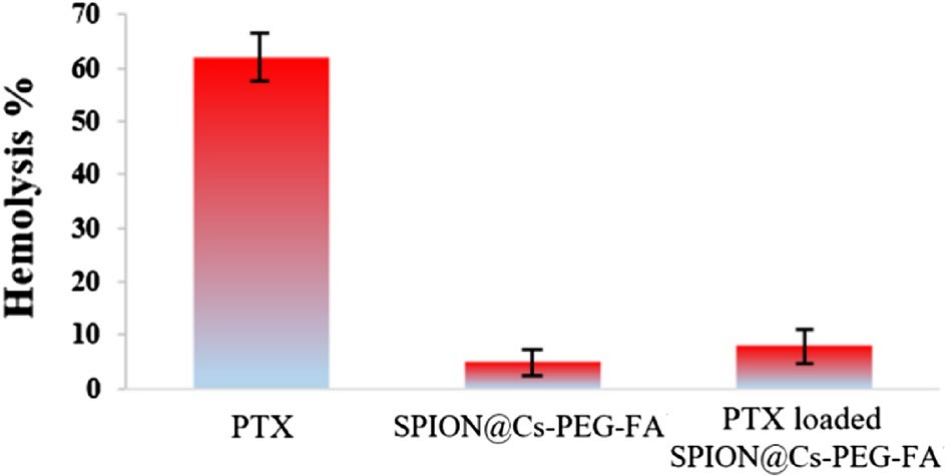
Quantitative hemolysis rates of PTX, SPION@Cs-PEG-FA, and SPION@Cs-PTX-PEG-FA. The error bars represent ± s.d. from n = 3.

Blood compatibility is one of the important challenges in in vivo applications. Consequently, a hemolysis test was performed to prove the hemocompatibility of SPION@Cs-PTX-PEG-FA. As shown in Fig. 17, erythrocytes exposed to PTX released ~ 62% of hemoglobins, showing that the reagents to solubilize PTX were significantly toxic. However, PTX, SPION@Cs-PEG-FA and SPION@Cs-PTX-PEG-FA indicated a low hemolytic amount in erythrocytes compared to free PTX (Fig. 17). Finally, the result showed that SPION@Cs-PTX-PEG-FA was suitable for nanoformulations for in vivo therapeutic applications.

### Effect of SPION@ Cs-PTX-PEG-FA on tumor development and survival rate

**Figure 18:**
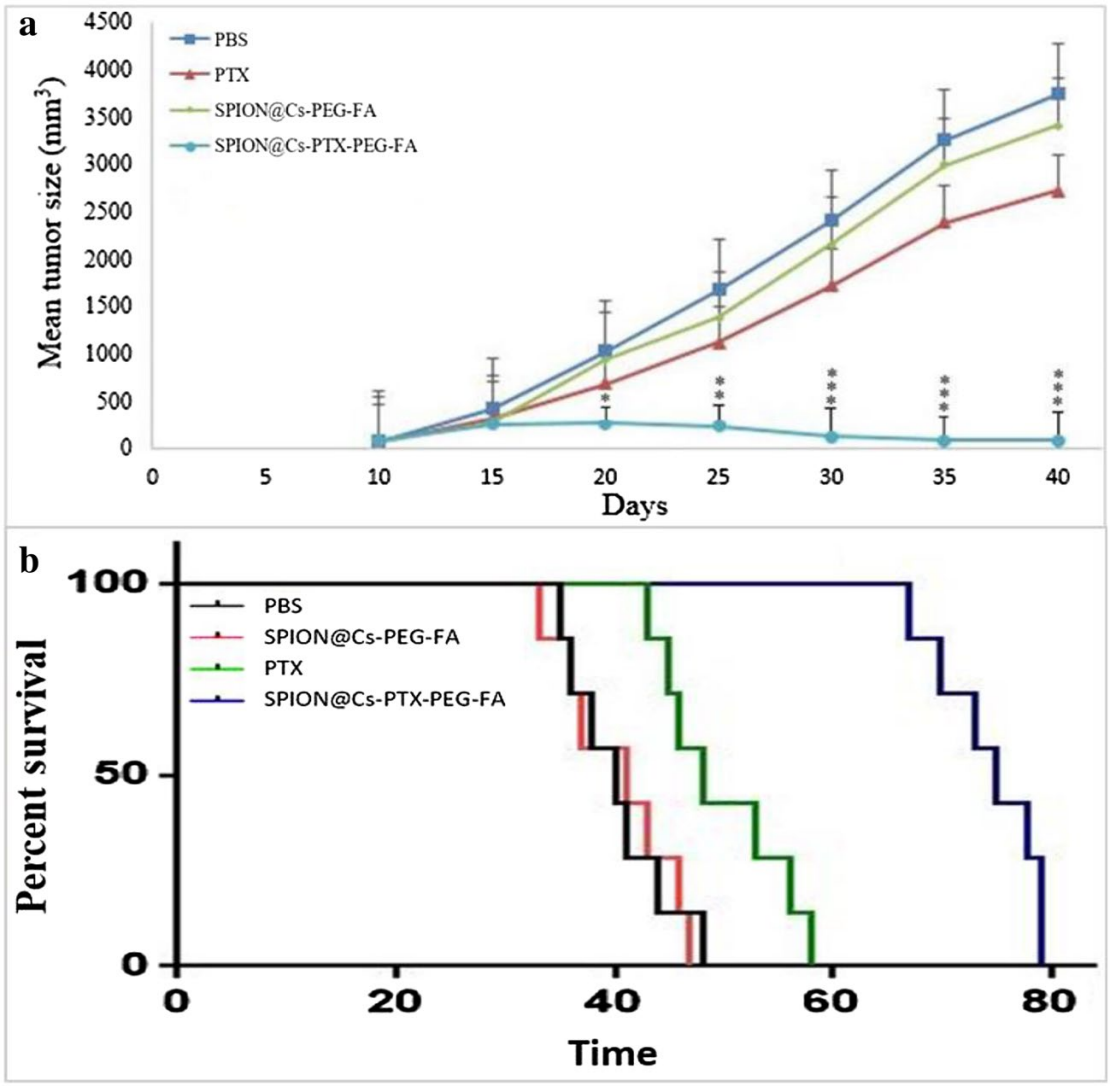
In vivo antitumor effects of SPION@Cs-PTX-PEG-FA in a mouse tumor model. Changes in tumor size were observed in representational mice (four groups). On day 19, tumor growth in mice treated with SPION@Cs-PTX-PEG-FA was reduced significantly (the values in the graph indicate the mean ± SD; *p < 0.05, **p < 0.01 and ***p < 0.001, represent significant differences between the control (untreated) and other treatments) relative to that of the other groups that received free PTX, SPION@Cs-PEG-FA, and PBS.

In vivo antitumor efficacy of SPION@Cs-PTX-PEG-FA, free PTX, SPION@Cs-PEG-FA, and PBS were evaluated in female BALB/c mice. The time-dependent changes in tumor volume were plotted and are presented in Fig. 18a. The results showed that on day 19, tumor volume and growth were significantly inhibited (p < 0.01) inhibited in mice treated with SPION@Cs-PTX-PEG-FA compared to other groups incubated with empty PTX, SPION@Cs-PEG-FA and PBS. As shown in Fig. 18a, PTX did not significantly inhibit the growth of xenografts. On the contrary, SPION@Cs-PTX-PEG-FA had a profound inhibitory effect on the growth of these xenografts. Even at the end of the treatment, SPION@Cs-PTX-PEG-FA could efficiently keep the increased ratio of tumor at a stable and low level compared with controls (PBS and SPION@Cs-PEG-FA groups), Meanwhile, Fig. 18b shows that mice treated with SPION@Cs-PTX-PEG-FA were still alive by day 79 compared with those in the PTX, SPION@Cs-PEG-FA, and PBS groups that succumbed by days 58, 48, and 47, respectively (p < 0.05). Two distinct trials both produced results that were relatively similar to each other. We expanded our research to include BALB/c models of fibrosarcoma tumors and found that SPION@Cs-PTX-PEG-FA had a considerable antitumor impact in cancer cells, but none in normal cells. Even at large doses, PTX was safe and well tolerated by animals and humans^88^. However, the compound was able to reach only the surface of epithelial cells in the intestine and colon because it was poorly absorbed by the intestinal tract and quickly eliminated by chemical changes in the liver^89–91^.

### Measurement of lymphocyte proliferation index after treatment

**Figure 19:**
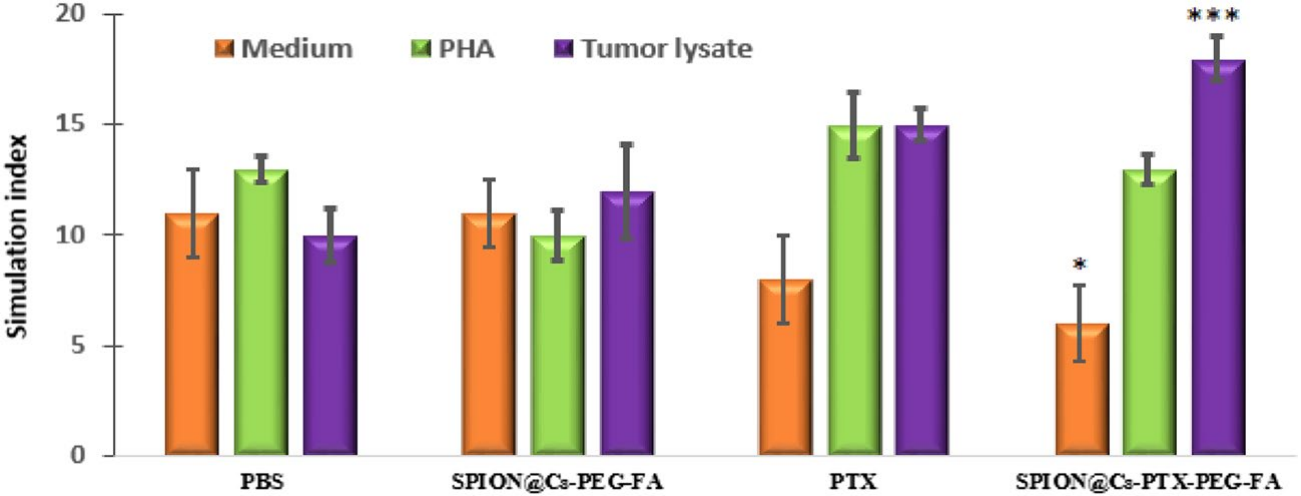
The proliferation index of splenocytes after induction of tumor lysate, PHA (positive control), and medium (negative control) was observed in mice receiving SPION@Cs-PTX-PEG-FA nanoformulation compared to those given PTX, SPION@Cs-PEG-FA, and PBS. The results indicate the mean of the measurements performed in triplicate (mean ± SD (*p < 0.05; **p < 0.01; ***p < 0.001); n = 3).

Following the administration of tumor lysate and PHA (the positive control), the proliferation index of splenocytes was determined in the spleen tissues of the four mouse groups. Figure 19 shows the proliferation rates of splenocytes taken from mice that received SPION@Cs-PTX-PEG-FA. The rates were statistically and significantly higher than those taken from the other three groups. When stimulated with either PHA or medium, splenocytes did not show statistically significant variations in their proliferation rates between groups. Other studies have shown a strong link between the antitumor effect of PTX-loaded polymeric nanocarrier and immunological responses, such as splenocyte growth and cytokine production. These results were in line with those of those other studies^61,92^.

### Measurement of IFN-γ and IL-4 levels after treatment

**Figure 20:**
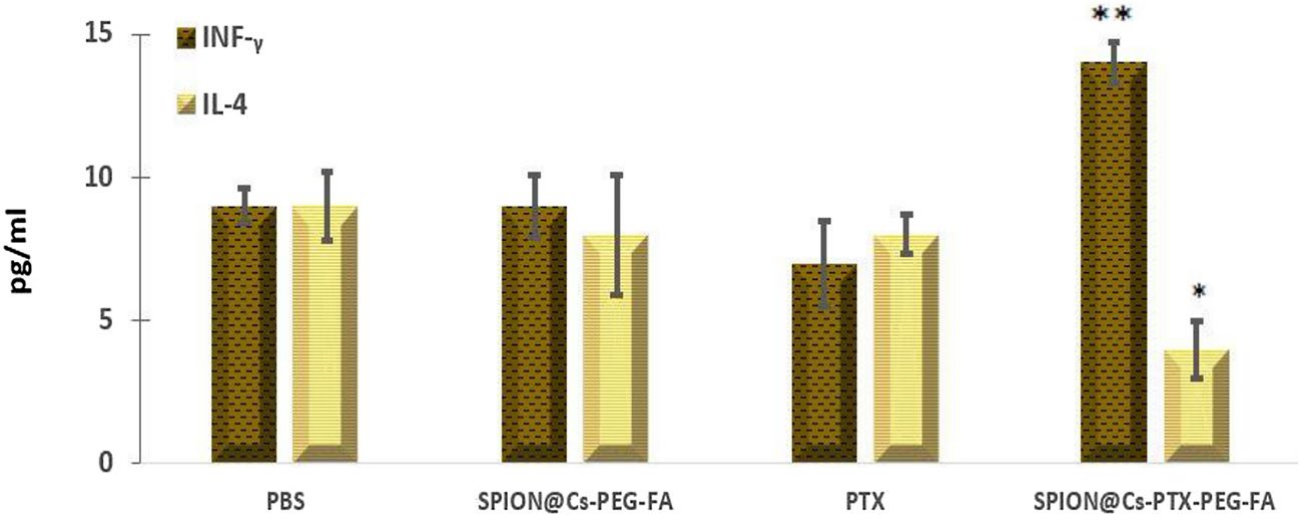
Splenocytes collected from mice treated with SPION@Cs-PTX-PEG-FA were restimulated using tumor lysate. A substantial increase (p < 0.05) in IFN-γ levels compared to those of the other groups and a significant decrease (p < 0.05) in IL-4 production were observed in mice receiving SPION@Cs-PTX-PEG-FA nanoformulation compared to those receiving PTX, SPION @ Cs-PEG-FA and PBS. The results indicate the mean of the measurements performed in triplicate (mean ± SD (*p < 0.05; **p < 0.01; ***p < 0.001); n = 3).

Splenocytes obtained from mice treated with SPION@Cs-PTX-PEG-FA showed a significant (p 0.05) increase in IFN-γ level when restimulated with tumor lysate compared to splenocytes obtained from mice in the other groups, although the overall level of IFN-γ was slightly low in all samples. The experimental results are shown in Fig. 20. Furthermore, the amount of IL-4 produced by mice who received SPION@Cs-PTX-PEG-FA was significantly reduced (p 0.05), compared to the amount of IL-4 produced by animals who received PTX dendrosome and PBS. There were no discernible differences between the groups based on the results obtained from the samples stimulated with PHA (the positive control) and medium (the negative control) (data not shown). IFN-γ expression levels were correlated with alterations in cytokine generation, such as antitumor response^93–95^.

## Conclusions

Targeting mechanisms can be challenged by the stability of nanomaterials, with the release of pH-dependent medication, SPION@Cs-PTX-PEG-FA NPs demonstrated novel biological activity. These NPs can serve as a PTX pharmacological magnetic vehicle with SPION@Cs-PTX-PEG-FA relatively modest cytotoxic effects. Furthermore, in vivo findings demonstrated that tumor formation decreased substantially (p < 0.01) decreased in mice treated with SPION@Cs-PTX-PEG-FA compared to animals given free PTX. Therefore, SPION@Cs-PTX-PEG-FA can be developed into a novel formulation to significantly enhance the synergistic therapeutic effects of magnetic drug nanocarriers by binding with the anticancer drug PTX. This formulation may be used in the future clinical treatment of cancer.

## Data Availability

The datasets generated and/or analyzed during the current study are available from the corresponding author (N. A) on reasonable request.
